# Ion Compensation-Assisted Photolithography Enables High-Resolution Electrolytes for Neuromorphic Transistors

**DOI:** 10.1007/s40820-026-02288-4

**Published:** 2026-07-13

**Authors:** Wenjing Zhang, Xiaoci Liang, Sixing Chen, Xiuquan Ma, Chen Chen, Songjia Han, Chuan Liu

**Affiliations:** 1 State Key Laboratory of Optoelectronic Materials and Technologies and Guangdong Province Key Laboratory of Display Material and Technology, School of Electronics and Information Technology, Sun Yat-Sen University, Guangzhou, 510275 People’s Republic of China; 2 College of Artificial Intelligence and Low-Altitude Technology, South China Agricultural University, Guangzhou, 510642 People’s Republic of China; 3 Science and Technology on Advanced Ceramic Fibers and Composites Laboratory, College of Aerospace Science and Engineering, National University of Defense Technology, Changsha, 410073 People’s Republic of China; 4 Guangdong Provincial Key Laboratory of Digital Manufacturing Equipment, Guangdong HUST Industrial Technology Research Institute, Dongguan, 523000 People’s Republic of China

**Keywords:** Organic electrochemical transistors, Photolithography, Electrolyte, Synaptic transistor

## Abstract

High-density organic electrochemical transistor (OECT) arrays are essential for neuromorphic computing and bioelectronic interfaces, but progress has been limited by the low resolution of electrolyte patterning. Although conventional photolithography offers high feature resolution, it involves a fundamental trade-off among spatial resolution, ionic capacitance, and stability in the electrolyte. Here we report an ion compensation-assisted photolithography (ICAP) strategy that yields electrolyte micro-patterns combining high precision, high capacitance and high stability. A molecularly engineered electrolyte forms, under UV exposure, a physicochemical dual cross-linked network with strong solvent resistance and hydrophobicity, which suppresses swelling during both aqueous development and the subsequent ion-compensation step, preserving pattern fidelity. Ion compensation then restores and enhances the mobile-ion content, increasing areal capacitance. The resulting electrolytes achieve a record 2 μm resolution, 15.6 μF cm^−2^ capacitance, and strong thermal stability from − 50 to 200 °C. Integrated into OECTs, the ICAP-patterned electrolytes suppress crosstalk by 97.6% and boost on/off ratios by 325%, reducing parasitic coupling by more than 40 times compared to unpatterned arrays. The method is compatible with *p*-type and *n*-type organic semiconductors and inorganic oxides, providing a versatile route to scalable neuromorphic circuits and advanced bioelectronics.

**Supplementary Information:**

The online version contains supplementary material available at 10.1007/s40820-026-02288-4.

## Highlights

- Ion compensation–assisted photolithography (ICAP) combines UV-triggered dual crosslinking with a post-patterning ion replenishment step, enabling patterned electrolytes with a spatial resolution of 2 μm, a capacitance of 15.6 μF cm^−2^.
- ICAP-patterned electrolytes provide array-level ionic isolation by spatially confining ion transport, suppressing lateral ionic leakage and inter-device crosstalk in organic electrochemical transistor arrays.
- ICAP process is broadly compatible with *p*-type PEDOT:PSS, *n*-type benzimidazo-benzophenanthroline, and inorganic WO_3_, establishing a versatile electrolyte-patterning strategy for high-density electrolyte-gated devices.

**Supplementary Information:**

The online version contains supplementary material available at 10.1007/s40820-026-02288-4.

## Graphical abstract

**Figure:**
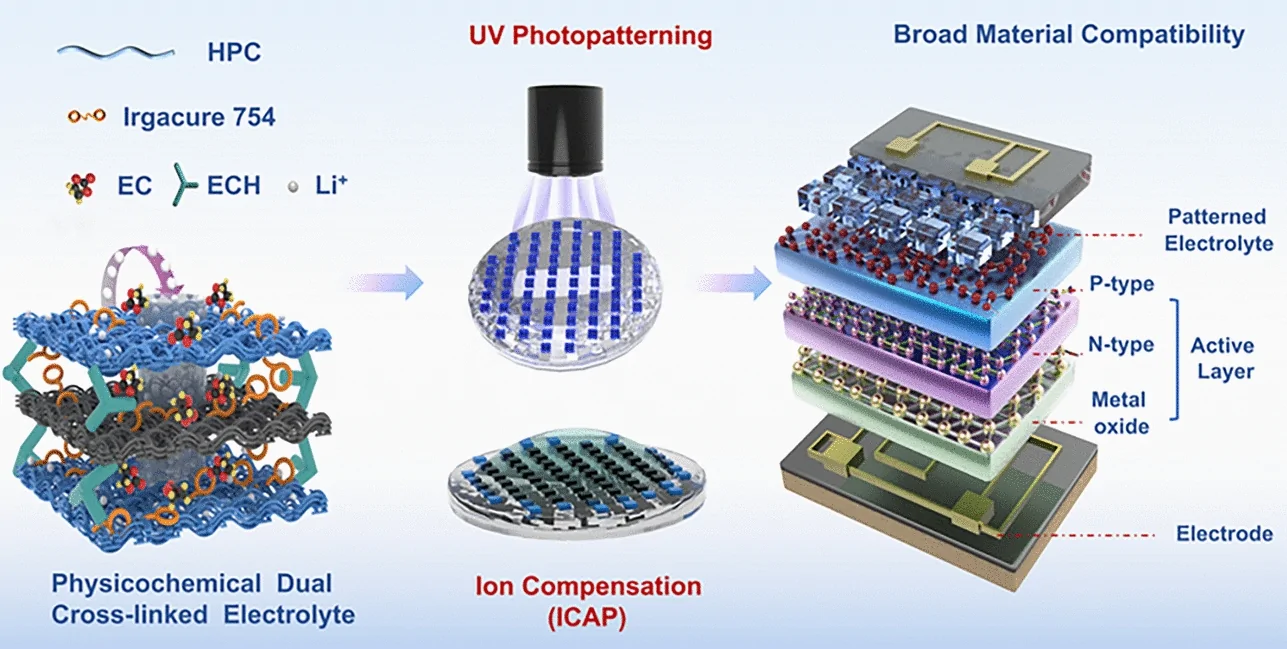

## Introduction

Organic electrochemical transistors (OECTs) have emerged as a distinctive class of transistors that combine low-voltage operation, high transconductance, flexibility, and biocompatibility [1]. These unique advantages make OECTs highly promising for applications ranging from neuromorphic computing to bioelectronic interfaces and flexible integrated circuits [2–4]. Conductivity modulation is achieved through the electrolyte-to-channel ion injection, which dynamically alters the doping state of the semiconductor [5, 6]. The very property that enables OECTs, their dependence on mobile ionic electrolytes, also introduces significant challenges for miniaturization and large-scale integration. The presence of unpatterned electrolyte in densely packed arrays can degrade the on/off ratio, increase power consumption, and induce severe inter-device crosstalk due to parasitic ionic currents and capacitive coupling [7]. To fully exploit their properties in large-scale integrated circuits, OECT arrays need to achieve high integration density while maintaining robust electrical isolation. However, recent research has predominantly focused on single-OECT architectures, and the inability to precisely pattern electrolytes has critically limited the development of scalable, integrated circuits based on high-density OECT arrays [8, 9].

Multiple strategies have been explored to address this issue. Printing-based techniques such as screen printing, inkjet printing, self-assembly, and transfer printing allow straightforward electrolyte patterning, yet they suffer from limited resolution, low yield, and poor throughput [10–14]. Standard mask-based photolithography has recently enabled tens-of-micrometer-scale patterned electrolyte features by using photo-assisted crosslinking to form an insoluble resist-like network upon UV exposure [15–17]. However, the continued shrinking of electrolyte dimensions imposes stringent requirements on mobile-ion density, because micropatterned electrolytes must retain a high capacitance to drive OECT operation. In conventional photolithographic approaches, the highly cross-linked networks needed to preserve high-resolution patterns often restrict mobile-ion content and transport, thereby reducing ionic conductivity and capacitance. While increasing the ion concentration in the precursor formulation can improve capacitance, it generally compromises the mechanical integrity of the patterned electrolyte and makes precise lithographic definition more challenging [18–20]. Moreover, beyond the need to simultaneously achieve high patterning resolution and high capacitance, temperature stability also represents a critical requirement for practical device operation. Conventional hydrogel-based electrolytes offer excellent ionic transport and high capacitance but suffer from rapid water loss at small scales, leading to structural instability and device degradation. In contrast, polyelectrolytes, though more stable, exhibit slow ion mobility, resulting in hysteretic behavior and limited response speed [16, 21]. Thus, the trade-off among high resolution, high capacitance, and high stability arises not only from intrinsic material limitations, but more fundamentally from the incompatibility between traditional patterning processes and the ionic functionality required for electrolyte-gated operation. To date, no electrolyte has been reported that simultaneously satisfies the requirements for both high capacitance and stability under miniaturized conditions [22, 23].

Here, we introduce an ion compensation-assisted photolithography (ICAP) strategy to address the long-standing trade-off among patterning resolution, ionic capacitance, and operational stability in miniaturized electrolytes. The key conceptual advance of ICAP is that it decouples two requirements that are usually incompatible in conventional electrolyte patterning: precise structural definition during photolithography and high mobile-ion content for effective electrolyte gating. To implement this concept, we developed a photolithographic electrolyte (PLE) that forms a solvent-resistant physicochemical dual cross-linked network upon UV irradiation. This network enables accurate photopatterning with minimal swelling, while the subsequent ion-compensation step replenishes mobile ions after lithographic fixation, improving the capacitance of the electrolyte without compromising structural integrity. Using ICAP, we achieve patterned electrolytes with a spatial resolution of 2 μm, a large capacitance of 15.6 μF cm^−2^, and exceptional stability across an ultrawide temperature range (− 50–200 °C). When integrated into OECTs, the well-defined ICAP-electrolytes boost the on/off ratio by 325% and suppress inter-device crosstalk by 97.6% compared to unpatterned counterparts. The ICAP-patterned electrolyte is broadly compatible with *p*-type and *n*-type organic semiconductors and inorganic oxide semiconductors, underscoring its versatility across diverse material systems. The ICAP strategy provides a scalable platform for high-performance electrolyte-gated transistors and neuromorphic electronics, providing a viable route to high-density, wafer-scale hybrid integrated OECT-based electronics.

## Experimental Section

### Materials

Hydroxypropyl cellulose (HPC), lithium chloride (LiCl, ≥ 99.8%), ethylene carbonate (EC), epichlorohydrin (ECH), and tungsten hexachloride (WCl_6_) were obtained from Aladdin. benzimidazo-benzophenanthroline (BBL), methanesulfonic acid (MSA), and Irgacure 754 were sourced from Sigma-Aldrich. Poly(3,4-ethylenedioxythiophene)- poly(styrenesulfonate) (PEDOT:PSS) was acquired from Heraeus Germany. Dimethyl sulfoxide (DMSO) was purchased from Macklin Biochemical Co., Ltd., Methylene blue was purchased from Tokyo Chemical Industry.

### Fabrication of ICAP-Patterned Electrolytes and OECTs

#### PLE Precursor Preparation

First, HPC (125 mg mL^−1^) and LiCl (5 mg mL^−1^) were dissolved in an aqueous mixture of EC and water (10:90, w/w). The mixture was stirred at 90 °C for 2 h and then cooled to room temperature to yield the HPC/EC solution. Separately, Irgacure 754 was diluted with an equal volume of ethanol. Subsequently, the HPC/EC solution, Irgacure 754 solution, and ECH were mixed in a volume ratio of 2:7:1. The mixture was vortex-mixed for 30 s to obtain a homogeneous, optically transparent precursor solution.

#### Standard Mask-Based UV Photolithography

Polyethylene terephthalate (PET) or silicon wafer substrates were treated with vacuum plasma (SUNJUNE PLASMA PT-5S) to enhance surface hydrophilicity. Then, the PLE precursor was spin-coated onto the substrate at 2000 rpm for 30 s. The film was then subjected to a two-stage annealing (125 °C for 5 min and 85 °C for 40 min). Given the higher volatility of water compared to the high-boiling-point EC, this treatment only removes the water, preserving EC within the polymer matrix to function as the plasticizing phase. Subsequent cooling to room temperature solidified the EC, yielding a solid film. The samples were then exposed to ultraviolet light (SEN PL17-110) through a quartz mask for 5 min, followed by development in water to yield patterned structures. The patterned electrolytes were immersed in LiCl solutions (10, 30, and 50 mg mL^−1^) for 4 h to enhance their capacitance. The residual LiCl solution was removed by blowing with a N_2_ gun.

#### Fabrication of *P*-type OECTs

The PET substrate was subjected to plasma treatment to enhance surface hydrophilicity. Subsequently, source, drain, and gate electrodes (5 nm Cr, 50 nm Au) were deposited via thermal evaporation, with channel dimensions defined as 50 μm in length and 1000 μm in width. PEDOT:PSS doped with 10 wt% DMSO was printed using a Sonoplot Microplotter II system. The printed film was then annealed at 100 °C for 15 min to remove residual solvents. The average thickness of the PEDOT:PSS was 347 nm. Finally, the PLE layer was patterned via ICAP process.

#### Fabrication of *N*-type OECTs

The glass substrate was sequentially cleaned by ultrasonication in isopropanol, ethanol, and deionized water for 10 min each, followed by nitrogen gas drying. Source, drain, and gate electrodes were then deposited via thermal evaporation, with channel dimensions defined as 50 μm in length and 1000 μm in width. A hydrophobic organic solution was spin-coated onto the substrate at 2000 rpm for 30 s, and annealed at 100 °C for 10 min to remove residual solvents, forming a hydrophobic film. Thereafter, the substrate, covered with a shadow mask, was then treated with 70 W oxygen plasma etching for 300 s. BBL solution (5 mg mL^−1^ in MSA) was spin-coated at 2000 rpm for 30 s. The sample was immersed in DI water for 4 h to remove the MSA, followed by annealing on a hot plate at 140 °C for 30 min. A uniform BBL film with an average thickness of 71 nm was obtained. Finally, the PLE layer was patterned via ICAP process to yield the BBL-OECT.

#### Fabrication of WO_3_-OECTs

The WO_3_-OECTs were fabricated on Si/SiO_2_ wafers. The substrate cleaning procedure was identical to that described for glass substrates. Gate, source, and drain electrodes (5 nm Cr, 50 nm Au) were deposited onto the substrate via thermal evaporation. 0.1 mol L^−1^ WCl_6_ precursor solution was prepared and spin-coated at 4000 rpm for 30 s. Then, the sample was annealed in air at 200 °C for 10 min, followed by 400 °C for 1 h, yielding a WO_3_ film with an average thickness of 28 nm. The WO_3_ channel was patterned by dry etching using SF_6_ and O_2_. The PLE was subsequently deposited and patterned to produce the WO_3_-OECT.

### Characterization

The microstructure of the patterned electrolyte was examined using a polarization microscope (Leica DM2700P). The infrared spectra of the synthesized materials were analyzed by Fourier-transform infrared spectroscopy (FTIR, Thermo Fisher Nicolet iS5). X-ray photoelectron spectroscopy (XPS) measurements were carried out by Thermo Scientific ESCALAB Xi + . The structure and elemental composition of the patterned electrolyte were analyzed using a scanning electron microscope (SEM, Carl Zeiss, SUPRA 60). The physical properties of the materials were evaluated using differential scanning calorimetry (DSC, Netzsch 204 F1) and thermogravimetric analysis (TGA, Netzsch TG209F1 Libra). The electrical properties of the OECTs were assessed with a Semiconductor Parameter Analyzer (PDA FS Pro). The thermal endurance of the devices was evaluated by electrical characterization using a temperature-controlled hot plate in combination with a Semiconductor Parameter Analyzer [24]. Electrochemical properties were investigated using an electrochemical workstation (CHI660E, Shanghai Chenhua) with an applied alternating current voltage of 5 mV and a scanning frequency range of 10^5^ to 1 Hz. Contact angles of the electrolytes were quantitatively analyzed using a contact angle goniometer (Data Physics OCA 15 EC).

### Computational Details

#### PPF Measurement

Applying two sequential voltage pulses, PPF was defined as *A*_2_/*A*_1_, where *A*_1_ and *A*_2_ are the peak *I*_DS_ of the first and second pulses, respectively. The PPF index as a function of inter-pulse interval follows a double-exponential decay:

1 $${\mathrm{PPF}} = C_{0} + C_{1} \exp \left( { - \frac{\Delta t}{{\tau_{1} }}} \right) + C_{2} \exp \left( { - \frac{\Delta t}{{\tau_{2} }}} \right)$$

where ${C}_{1}$ and ${C}_{2}$ denote the slow and rapid phases, respectively. The pulse interval is denoted as Δt,$while {\tau}_{1}$ and ${\tau}_{2}$ represent the PPF relaxation times for the slow and rapid phases, respectively.

#### Dots Per Inch (dpi) Measurement

The dpi of the patterned electrolyte is calculated based on the number of electrolyte features (e.g., dots, lines) deposited within a unit length of the substrate. The formula is given by:

2 dpi=N/L

where *N* denotes the total number of electrolytes counted along a specified direction and *L* represents the corresponding physical length in inches.

#### TCAD Simulation

Two-dimensional TCAD simulations were performed to compare OECT structures with unpatterned and patterned electrolyte configurations. The simulated model consisted of a semiconductor channel, source/drain electrodes, a gate electrode, and an electrolyte region.

Ion transport in the electrolyte was described by the Nernst–Planck drift–diffusion equation coupled with the Poisson equation:

3 $$\frac{\partial {c}_{i}}{\partial t}=-\nabla \cdot {J}_{i}$$

4 $${J}_{i}=-{D}_{i}\nabla {c}_{i}-{z}_{i}{\mu}_{i}q{c}_{i}\nabla \phi$$

5 $$\nabla \cdot (\varepsilon \nabla \phi )=-\rho$$

where ${c}_{i}$, ${D}_{i}$, ${\mu}_{i}$, and ${z}_{i}$ are the concentration, diffusion coefficient, mobility, and charge number of mobile ion species i, respectively; ϕ is the electrostatic potential, q is the elementary charge, and ε is the permittivity. The electronic transport in the semiconductor was described using the semiconductor drift–diffusion model. The detail material and structure parameters are summarized.

## Results and Discussion

### Resolving the Resolution–Capacitance Trade-off via ICAP Electrolytes

**Fig. 1:**
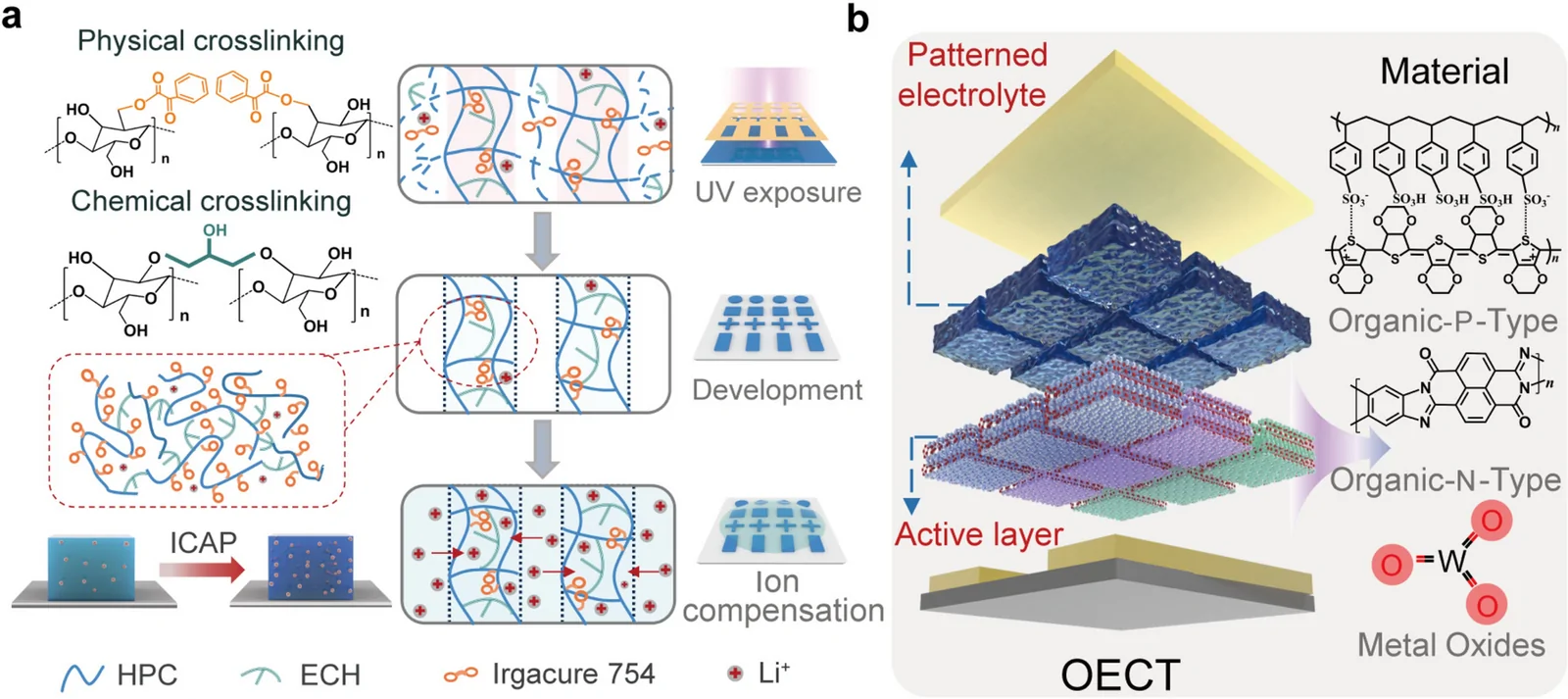
Fabrication and basic characteristics of ICAP-patterned photolithographic electrolytes (PLEs). **a** Schematic of ICAP-patterned electrolyte fabrication. **b** Compatibility of aqueous development with organic and inorganic semiconductors

A persistent challenge in integrating OECTs into high-density circuits is the long-standing trade-off between patterning resolution, ionic capacitance, and stability of the electrolytes, which fundamentally constrains device miniaturization and performance. To overcome this limitation, we developed an ion compensation-assisted photolithography (ICAP) strategy, which integrates UV-triggered dual physicochemical crosslinking with a post-patterning ion replenishment step. In Fig. 1a, the thick blue lines denote the HPC chains, which serve as the backbone of the electrolyte matrix. Upon UV irradiation, radicals generated from Irgacure 754 become covalently grafted onto the HPC chains, introducing hydrophobic moieties into the polymer structure. These hydrophobic moieties subsequently induce reversible non-covalent associations within the polymer matrix, including hydrophobic interactions and chain entanglement, forming a hydrophobically associated physical crosslinking network [25]. This physical crosslinking network enhances the swelling resistance, structural stability, and pattern fidelity of the electrolyte. Concurrently, ECH reacts with the hydroxyl groups of HPC, forming chemical crosslinks via nucleophilic substitution, thereby establishing a dual physicochemical network with enhanced solvent resistance and hydrophobicity [26]. This robust network allows the patterned films to be processed entirely in water, enabling high-resolution patterning without the need for additional photoresists or dry etching steps. The patterned electrolyte can then be immersed in a concentrated LiCl solution for enhanced electrical performance, achieved through ion compensation. Thus, the combined effect of dual crosslinking and ion-compensation ensures structural fidelity while maintaining high electrochemical performance, which is essential for scaling OECT arrays. This aqueous development, resist-free approach also avoids damage to active semiconductors, ensuring broad material compatibility across both organic and inorganic systems, such as PEDOT:PSS, BBL, and WO_3_ (Fig. 1b).

**Fig. 2:**
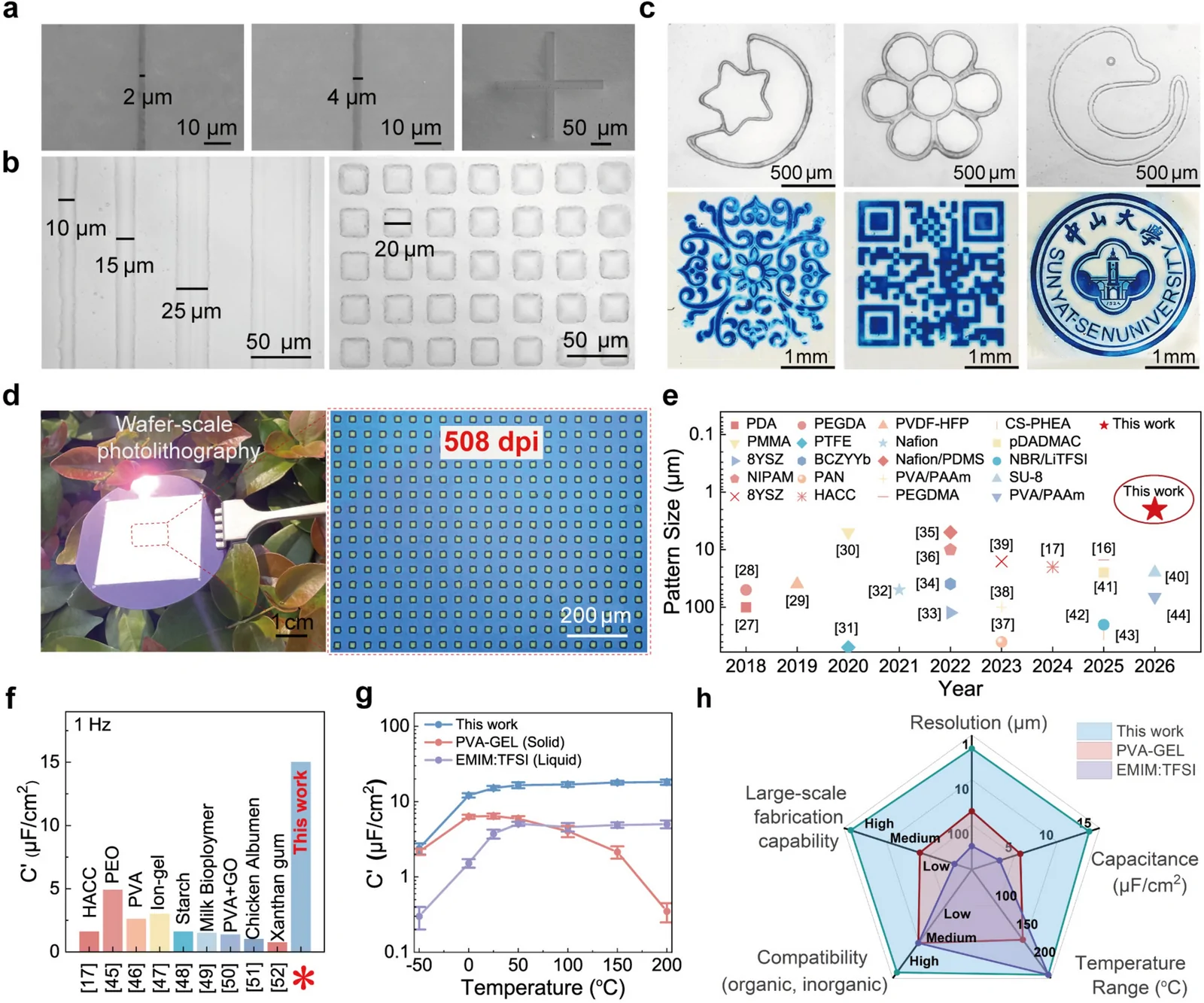
**a** SEM images and **b** OM images showing 2 μm resolution features. **c** OM images showing complex features. **d** Photograph of the wafer-scale fabrication of ICAP-patterned electrolyte array. **e** Benchmark comparison of electrolyte patterning resolution. **f** Benchmark comparison of electrolyte patterning capacitance. **g** Thermal stability (− 50 to 200 °C) of the capacitance. **h** Summary of multifunctional advantages: high resolution, capacitance, large-scale fabrication capability, compatibility and thermal tolerance

SEM images and optical microscopy (OM) images reveal that the patterned PLE supports minimum linewidths of 2 μm (Figs. 2a–c and S1). Demonstrating its scalability, we achieved wafer-level fabrication of a uniform electrolyte array at a high density of 508 dpi (Fig. 2d). These results position our technique among the highest resolution electrolyte patterning methods reported to date (Fig. 2e) [16, 17, 27–44]. Figure S2 shows an array-level demonstration of ICAP-patterned OECTs with various channel lengths, highlighting the importance of micrometer-scale electrolyte patterning for future highly integrated OECT arrays. By defining well-confined electrolyte regions, such patterning can suppress lateral ionic diffusion, parasitic ionic currents, and capacitive coupling between adjacent devices, thus enabling isolated electrolyte islands and narrow inter-device isolation gaps. These features are essential for reducing crosstalk in dense arrays.

Electrochemical impedance spectroscopy (EIS) confirms that the patterned PLE achieves a capacitance of 15.6 μF cm^−2^ (Fig. S3), significantly surpassing the reported solid-state electrolytes by 3–15-fold improvement (Fig. 2f) [17, 45–52]. To benchmark the ICAP-fabricated electrolyte against reported state-of-the-art electrolytes, key parameters including minimum feature size, capacitance, and ionic conductivity are summarized in Table S1. The ICAP-fabricated electrolyte shows a combined improvement in patterning resolution, capacitance, and ionic conductivity, highlighting the balanced and enhanced performance enabled by the ICAP method.

Moreover, the PLE maintains stable capacitance across an ultrawide thermal window from − 50 to 200 °C (Figs. 2g and S4), expanding the operational window by approximately 100 °C compared to typical hydrogel electrolytes (~ 167% of their window). This high thermal stability is attributed to the ethylene carbonate (EC)-plasticized matrix. EC’s high boiling point (~ 248 °C) limits solvent loss at elevated temperatures, while the dual cross-linked network retains mechanical integrity. To further evaluate device-level thermal robustness, we measured the transfer characteristics of ICAP electrolyte-based OECTs over a temperature range of 25 to 150 °C (Fig. S5). The OECTs were fabricated with PEDOT:PSS as the semiconductor on flexible PEN substrates. The devices retained gate modulation up to 125 °C, indicating that the ICAP electrolyte can support OECT operation under moderately elevated temperatures. At 150 °C, the transfer characteristics degraded with reduced initial channel current and weakened gate modulation. This degradation suggests that the thermal limit of the present OECT is governed by the full device stack rather than the electrolyte alone. Possible contributing factors include changes at the electrode/electrolyte interface, semiconductor layer, contact region, or bias-induced interfacial electrochemical reactions. Overall, these results demonstrate that ICAP provides a robust and scalable solution for electrolyte patterning and successfully addresses the long-standing trade-off among structural fidelity, electrochemical performance, and stability in photolithographic electrolytes. This combination of high resolution, capacitance, wafer-scale processability, material compatibility and thermal robustness have, to our knowledge, not been demonstrated together previously (Fig. 2h). This capability enables the monolithic integration of OECTs with electrochemical random-access memory (ECRAM), and/or CMOS, making feasible high-density, wafer-scale hybrid integrated circuits.

### Mechanistic Insights into Dual Crosslinking and Ion Compensation

**Fig. 3:**
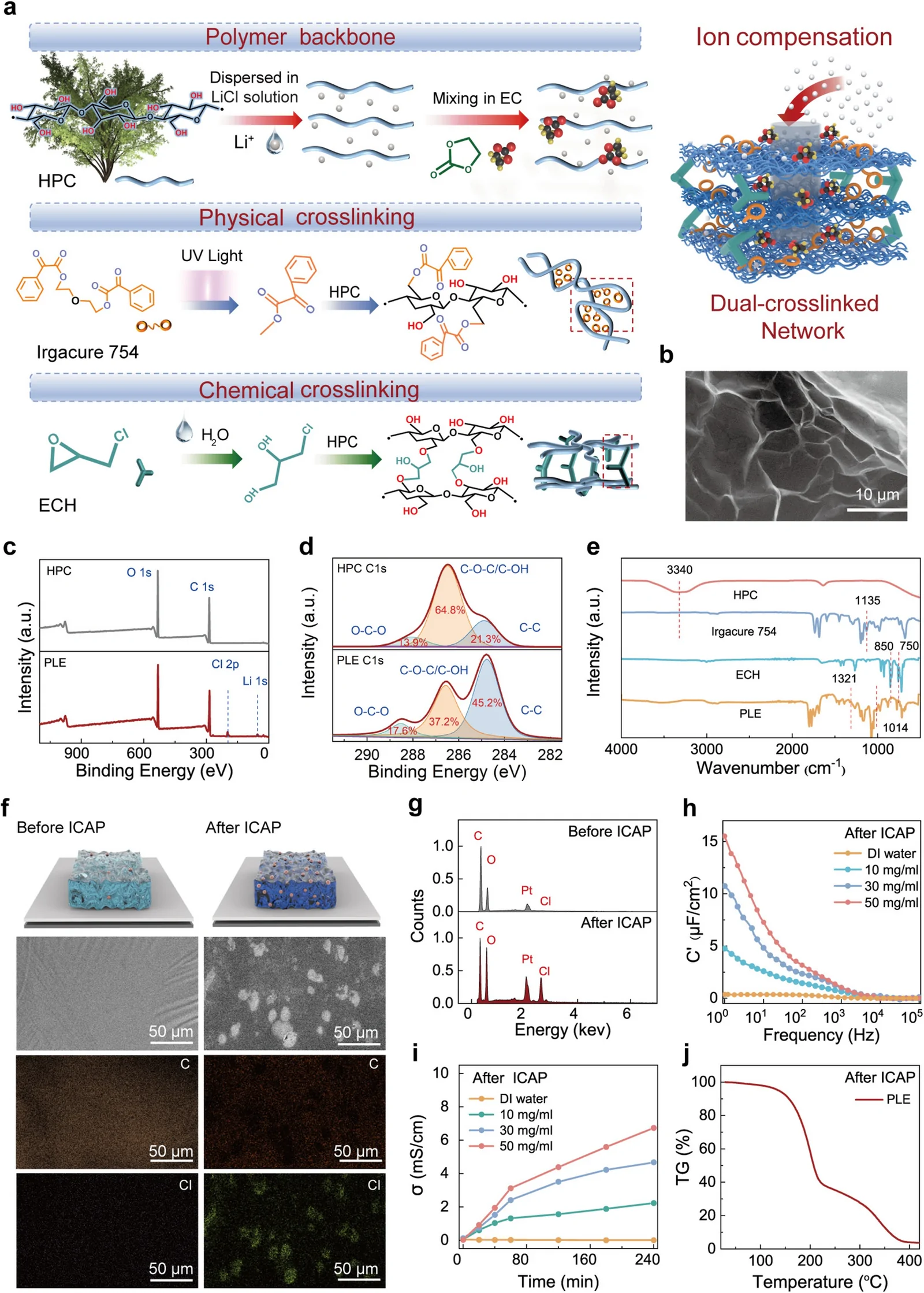
Mechanistic and spectroscopic evidence of PLE crosslinking and ion compensation. **a** Schematic of dual crosslinking mechanism: radical grafting via photoinitiator and etherification via epichlorohydrin. **b** SEM image of microporous structure facilitating ion transport. **c**, **d** XPS spectra showing hydroxyl consumption and ether bond formation. **e** FTIR spectra confirming epoxy ring opening and hydroxyl substitution. **f**, **g** EDS mapping and spectra before and after LiCl ion compensation. **h** Capacitance–frequency curves of PLEs treated with different LiCl concentrations. **i** Evolution of ionic conductivity during immersion in LiCl and DI water (different concentrations). **j** Thermogravimetric measurement demonstrating thermal stability up to 200 °C

To understand the molecular origin of the exceptional performance of ICAP-patterned electrolytes, we systematically investigated the underlying dual crosslinking chemistry and ion-compensation mechanism (Fig. 3a). The PLE leverages HPC as the structural backbone due to its biocompatibility and mechanical robustness, with EC selected as the solvent owing to its wide electrochemical stability window and high boiling point [53, 54]. Upon UV exposure, Irgacure 754 undergoes photolysis to generate methyl 2-oxo-2-phenylacetate radicals, which react with the hydroxyl groups of HPC to produce hydrophobic side chains [55, 56]. These side chains enhance in situ chain entanglement and induce physical crosslinking nodes (Fig. S6). At the same time, ECH undergoes nucleophilic substitution with the hydroxyl groups on HPC, initiating epoxide ring opening and forming additional chemical crosslinks [57]. As shown in Figs. 3b and S7, the cross-linked electrolyte exhibits an interconnected porous structure, with pore/free-volume features mainly distributed in the range of approximately 10–20 μm. Since these pore/free-volume features are much larger than solvated lithium ions, they present minimal resistance to ion migration. Therefore, the interconnected pore/free-volume features provide continuous ion-transport channels, allowing the electrolyte to maintain high ionic conductivity despite the presence of the cross-linked network. Moreover, during the ion-compensation process, the porous structure facilitates the diffusion of ions from the compensation solution into the interior of the gel electrolyte under a concentration gradient, thereby increasing the ion content throughout the electrolyte. Therefore, the microporous structure of the electrolyte enhances its electrochemical performance by preserving efficient ion transport and promoting effective ion compensation.

Spectroscopic analyses confirm the occurrence of these reactions. X-ray photoelectron spectroscopy (XPS) (Fig. 3c, d) reveals an enhanced intensity ratio of C–C (284.8 eV) to C–O (286.4 eV), consistent with hydroxyl group consumption via etherification [58]. Similarly, Fourier-transform infrared spectroscopy (FTIR) (Fig. 3e) shows a distinct reduction in the C–Cl stretching band (~ 750 cm^−1^) and the epoxy vibration (~ 850 cm^−1^), validating the epoxide ring-opening mechanism [59]. Additional O 1*s* XPS spectra (Fig. S8) demonstrate the appearance of C=O peaks at 531.5 eV relative to C–O–H at 532.5 eV, supporting the phenylacetate grafting hypothesis [60]. The disappearance of the O–H stretching band at 3340 cm^−1^ further corroborates hydroxyl substitution along the HPC backbone (Fig. 3e) [61]. The macroscopic film properties further reflect these chemical modifications. The contact angle of the PLE increases markedly from 16.3° to 62° (Fig. S9), indicating reduced surface hydroxyl content and enhanced hydrophobicity. Notably, the combined physical and chemical crosslinking network enhances the structural stability of the electrolyte films and suppresses their swelling during aqueous processing. As shown in Fig. S10, the patterned electrolyte retained clear boundaries and well-defined stripe structures after development and ICAP treatment, with no obvious delamination, peeling, or pattern collapse. The profilometer profiles further confirm that the electrolyte film underwent minor thickness variations throughout the process. In addition, after 24 h of immersion in DI water and subsequent water flushing, the patterned electrolyte remained intact without obvious deformation or pattern loss (Fig. S11). These results indicate that the cross-linked network endows the patterned electrolyte with sufficient structural and interfacial stability to withstand aqueous development and ICAP treatment.

During the compensation step, Li^+^ ions diffuse into the microporous matrix and form stable Li^+^–EC solvation complexes, effectively improving thermal and electrochemical stability [62]. Energy-dispersive X-ray spectroscopy (EDS) mapping (Figs. 3f and S12) reveals a significant enhancement of the Cl signal both on the electrolyte surface and across the electrolyte cross section after ion compensation. Although Li cannot be directly detected by EDS, the enhanced Cl signal serves as indirect evidence of LiCl incorporation, indicating that the compensation process occurs throughout the electrolyte film rather than being confined to the surface. Consistently, EDS and XPS analyses (Figs. 3g and S13, Tables S2 and S3) show a markedly increased Cl content after compensation, further supporting successful ion incorporation. Electrochemical impedance measurements reveal that immersion of the PLE in deionized (DI) water depletes mobile ions, leading to a marked reduction in capacitance and thus degraded electrochemical performance. In contrast, capacitance and conductivity increase steadily with increasing LiCl concentration (10, 30, and 50 mg mL^−1^) in the compensation solution and with immersion duration (Fig. 3h, i), indicating progressive ion uptake by the electrolyte. These findings demonstrate that the ICAP strategy not only restores the electrochemical performance of the patterned electrolyte but also enables continuous tuning of its capacitance by adjusting the concentration of the compensation solution. Finally, TGA/DSC were performed on the ion-compensated films (post-LiCl), which retain ≥ 90% mass up to 200 °C; capacitance–temperature measurements were taken on the same film, ensuring a like-for-like comparison (Figs. 3j and S14). In summary, dual crosslinking ensures structural robustness and localized ionic confinement, whereas ion-compensation restores and increases the mobile-ion content of the patterned electrolyte to enhance its electrochemical performance. By integrating these two mechanisms, the ICAP strategy enables a high-precision, high-capacitance patterned electrolyte, which is crucial for suppressing crosstalk and improving the on/off ratio in transistor arrays.

### Suppression of Leakage and Crosstalk in High-Density OECT Arrays

**Fig. 4:**
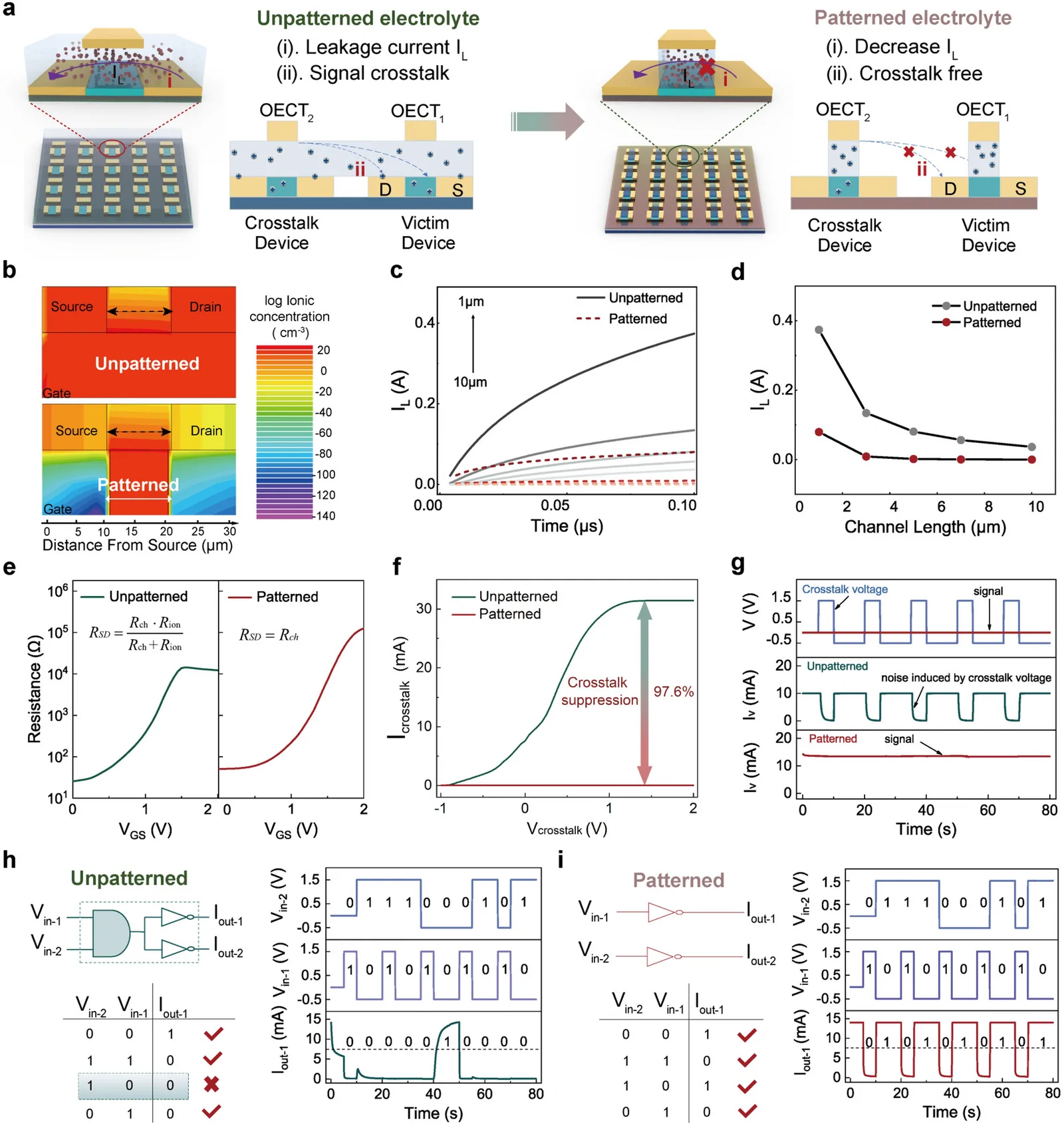
Suppression of leakage and electrical crosstalk in OECT arrays via ICAP electrolytes. **a** Schematics of unpatterned vs patterned OECT arrays. **b** TCAD simulations of ion distributions. **c**, **d** Leakage current (*I*_*L*_) as a function of channel length (simulated). **e** *R*_SD_–*V*_GS_ characteristics fitted with equivalent circuit models. **f** Crosstalk current (*I*_crosstalk_) versus crosstalk voltage (*V*_crosstalk_), showing 97.6% suppression in patterned arrays. **g** Transient crosstalk measurements for unpatterned vs patterned OECTs (vertical axis: the victim current I_v_), demonstrating suppression of crosstalk effect in the latter. **h**, **i** Pseudo-PMOS inverter operation: unpatterned devices exhibit NAND-like erroneous outputs, whereas patterned devices yield correct inverter logic with strong noise immunity

For practical integration of OECT arrays, suppressing leakage current and minimizing electrical crosstalk between neighboring devices is critical but remains challenging due to uncontrolled ion migration in unpatterned electrolytes. As illustrated in Fig. 4a, unpatterned OECT arrays allow mobile ions to migrate freely across adjacent transistors, which leads to substantial leakage current (*I*_L_) and strong inter-device coupling. These parasitic effects limit circuit scalability and result in unpredictable logic behavior. To overcome this limitation, we fabricated OECT arrays using ICAP-patterned electrolytes, which spatially confine ion transport and establish robust electrical isolation between neighboring channels. To clarify the role of electrolyte patterning in suppressing ionic crosstalk, two-dimensional TCAD simulations were performed to compare ion distribution and leakage-current pathways in OECT with continuous and patterned electrolytes (Fig. 4b, see Table S4 for details). For the continuous-electrolyte configuration, the electrolyte was modeled as an unpatterned film covering the channel region and overlapping with the source/drain electrodes. In contrast, for the patterned electrolyte configuration, the electrolyte was confined to the active channel region without direct contact with the source/drain electrodes, and the surrounding gap region was treated as ion-blocking. The simulations show that the continuous electrolyte leads to pronounced lateral ionic diffusion and leakage-current pathways, whereas the ICAP-patterned electrolyte effectively confines mobile ions near the addressed channel and suppresses leakage current. Additionally, as the channel length decreases (Fig. 4c, d), unpatterned devices exhibit sharply rising leakage currents, whereas patterned OECTs maintain consistently low I_L_ values, confirming the isolation effect of the patterned electrolytes.

To further verify the impact of leakage current and electrical crosstalk in practical devices, patterned and unpatterned OECT arrays were fabricated with PEDOT:PSS as the channel semiconductor. In unpatterned devices (Fig. 4e), large leakage currents introduce an additional ionic conduction pathway, such that *R*_SD_ reflects the combined contributions of the channel and electrolyte resistance (i.e., *R*_ch_ and *R*_ion_). This results in a non-monotonic *R*_SD_–*V*_GS_ dependence, showing an initial increase followed by a decrease as ionic conduction dominates. By contrast, ICAP-patterned devices effectively suppress *I*_*L*_, ensuring that *R*_SD_ is governed solely by the semiconductor channel. Consequently, the on/off ratio of patterned OECTs improves by 325% compared to unpatterned counterparts. Compared with previous strategies, ICAP suppresses leakage currents by spatially confining mobile ions without relying on encapsulation layers, whose complex processes often increase the difficulty of integrated circuit manufacturing [63, 64].

In addition, the crosstalk behavior of OECT arrays was quantitatively evaluated by biasing OECT_2_ as the aggressor gate while monitoring the parasitic current in the neighboring OECT_1_ (Fig. 4f). In unpatterned configurations, increasing the aggressor gate bias (*V*_crosstalk_) causes a substantial rise in the victim current (*I*_crosstalk_), defined as *I*_crosstalk_ = *I*_0_ − *I*, where *I*_0_ and *I* represent the initial and instantaneous currents of OECT_1_, respectively. This response originates from lateral ionic conduction and capacitive coupling within the shared electrolyte. In sharp contrast, ICAP-patterned arrays exhibit negligible parasitic response, achieving a 97.6% suppression of crosstalk under *V*_crosstalk_ = 1.5 V, corresponding to more than 40-fold reduction in parasitic coupling relative to unpatterned devices. Transient measurements further emphasize the benefits of electrolyte isolation (Fig. 4g). For unpatterned arrays, switching *V*_crosstalk_ between + 1.5 and − 0.5 V (with *V*_victim_ = 0 V) yields a signal-to-noise ratio (SNR) of − 7.75 dB, meaning that noise dominates the victim device response. Such negative SNR values indicate that inter-device crosstalk severely distorts signals and renders them indistinguishable from noise. In contrast, patterned arrays strongly suppress parasitic ionic currents, ensuring that the victim device remains electrically quiescent even under large aggressor gate swings, thereby preserving signal integrity.

The functional impact of ICAP is further demonstrated in pseudo-PMOS inverter circuits (Fig. 4h, i). In unpatterned arrays, parasitic ion capacitance through the shared electrolyte introduces wiring faults, producing NAND-like erroneous outputs instead of the expected inverter transfer characteristics. This malfunction arises because stray ionic coupling alters the effective gate bias of neighboring transistors, distorting logic thresholds and corrupting the truth table. In contrast, inverters constructed with ICAP-patterned OECTs generate correct logic outputs, reliably switching between logical “0” (*I*_out_ < 7.5 mA) and “1” (*I*_out_ > 7.5 mA) under input signals (*V*_in_ = 1.5 V/ − 0.5 V). These devices also exhibit strong immunity against crosstalk from adjacent inputs. These findings demonstrate that precise ionic confinement in ICAP-patterned electrolytes effectively suppresses leakage and minimizes crosstalk. This dual benefit directly addresses the fundamental limitations of OECT miniaturization, positioning ICAP as a practical route to scalable neuromorphic computing and logic circuits. By eliminating parasitic capacitive pathways while maintaining high ionic capacitance, ICAP provides a robust framework for the next generation of low-power, high-performance OECT electronics.

### Broad Material Compatibility and Neuromorphic Applications of ICAP Electrolytes

Beyond array-level integration, neuromorphic electronics demand electrolytes that are universally compatible with both organic and inorganic semiconductors. To assess this universality and reproducibility, we systematically evaluated the ICAP-patterned electrolyte across three representative semiconductors: *p*-type PEDOT:PSS, *n*-type BBL, and inorganic WO_3_. For each material system, five devices were independently fabricated and characterized. These systems cover the majority of OECT architectures, where ion capacitance critically governs performance and hybrid circuit functionality.

**Fig. 5:**
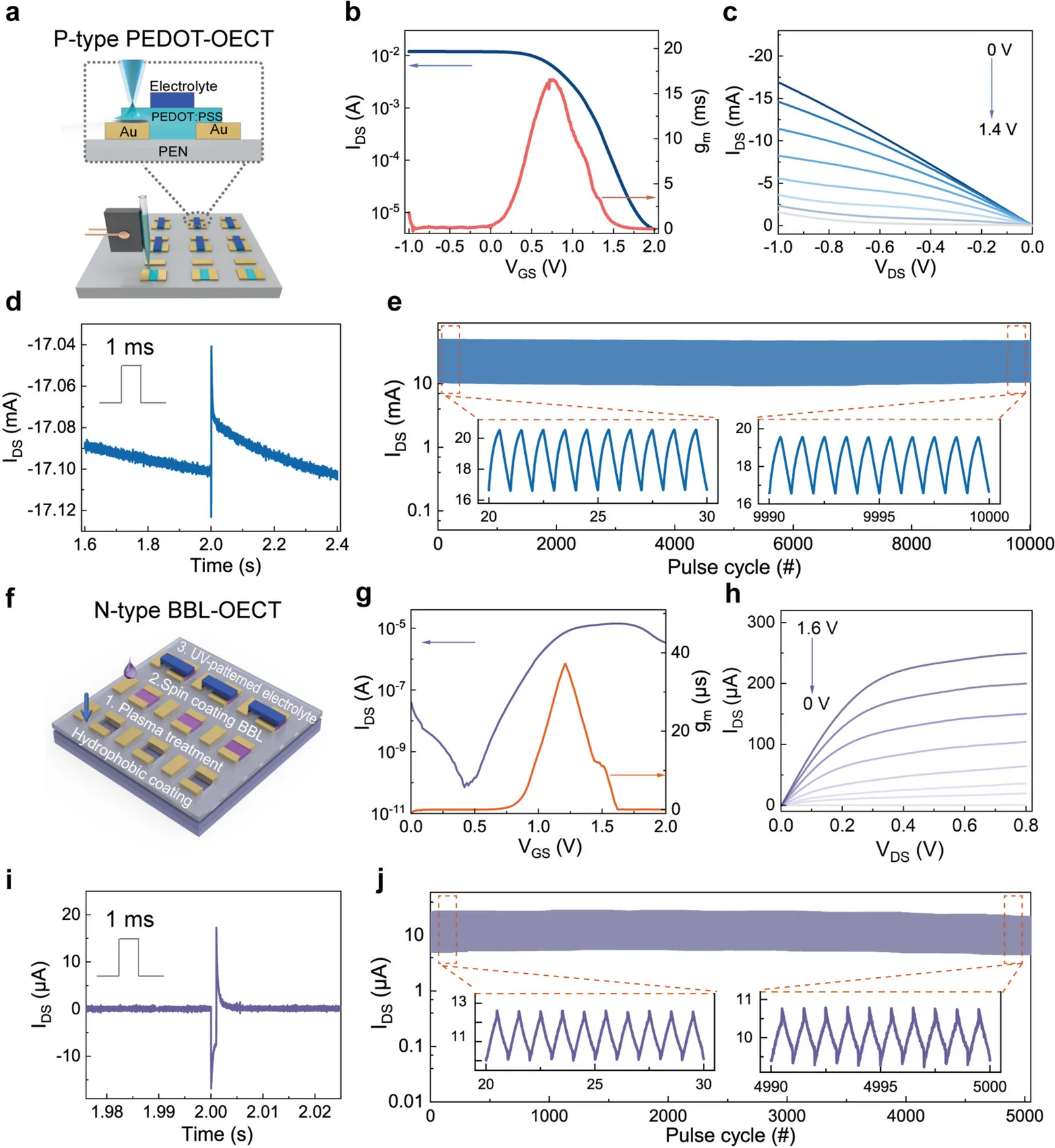
Compatibility of ICAP electrolytes with organic semiconductors and neuromorphic emulation. **a** Schematic of PEDOT-OECT fabrication. **b**, **c** Transfer and output curves demonstrating depletion-mode behavior with on/off ratio of 10^4^. **d** Inhibitory postsynaptic current (IPSC) responses showing millisecond-scale resolution and paired-pulse facilitation fitted with double-exponential function. **e** Long-term endurance with < 4.7% degradation after 10,000 cycles. **f** Fabrication schematic of BBL-OECT. **g**, **h** Transfer and output curves confirming accumulation-mode operation with on/off > 10^5^. **i** Excitatory postsynaptic current (EPSC) responses with 1 ms temporal resolution. **j** Endurance performance showing 85.3% current retention after 5000 cycles

For the *p*-type device, PEDOT:PSS films were precisely deposited using Sonoplot microplotter printing (Fig. 5a). The transfer and output characteristics (Fig. 5b, c) exhibit the expected depletion-mode behavior, where the drain current (*I*_DS_) decreases with increasing gate bias (*V*_GS_). Notably, with the ICAP-patterned electrolyte, PEDOT-OECTs achieve an on/off ratio of 10^4^ and an average transconductance of 16.5 ± 1.12 mS, nearly 10 times higher than conventional PEDOT:PSS OECTs (typically 10^2^ ~ 10^3^) [65]. This performance enhancement originates from the precise ionic confinement and high ionic capacitance in ICAP-patterned electrolytes, which suppress the off-state current and improves gate-channel coupling. Additionally, Fig. S15 shows the performance of the PEDOT-OECTs after bending. The devices were separately subjected to 200 bending cycles (bending radius of 5 mm) parallel and perpendicular to the channel length, after which the transfer characteristics remained comparable to those of the initial state. This result indicates that the ICAP-patterned electrolyte can maintain effective gate coupling under mechanical deformation. The bending stability is attributed to the balanced structure of the electrolyte: the dual cross-linked HPC framework provides sufficient structural integrity to suppress delamination or severe deformation. In addition, the patterned electrolyte may help reduce stress accumulation compared with a large-area continuous-electrolyte film. These results suggest that ICAP-patterned electrolytes possess promising mechanical robustness for flexible OECTs.

Beyond conventional transistor behavior, PEDOT-OECTs also emulate synaptic behaviors relevant to neuromorphic systems. In this configuration, the gate electrode functions as a presynaptic neuron, while the channel acts as the postsynaptic neuron. Applying a positive *V*_GS_ drives cations into the PEDOT:PSS film, generating an inhibitory postsynaptic current (IPSC). Upon bias removal, *I*_DS_ rapidly returns to baseline, demonstrating short-term plasticity (STP) with ~ 1 ms temporal resolution enabled by the efficient ion transport of ICAP-patterned electrolytes (Fig. 5d). The brief negative transient at the leading edge arises from capacitive displacement current and interfacial charging, a common feature in EGT/OECT dynamics. The IPSC increases with pulse intensity, resembling synaptic weight modulation in biological neurotransmission (Fig. S16a). Furthermore, when two sequential voltage pulses are applied (Fig. S16b), the second evokes a stronger response than the first, indicative of paired-pulse facilitation (PPF), where a fitted relaxation time of *τ*_1_ = 44.8 ms and *τ*_2_ = 679.3 ms, consistent with the reported biological synaptic dynamics. With an increasing number of pulses, *I*_DS_ gradually declines, marking the transition from STP to long-term plasticity (LTP) (Fig. S16c). Furthermore, the devices exhibit excellent operational endurance by retaining more than 95% of their initial current response after 10,000 continuous pulsing cycles as shown in Fig. 5e. This high level of cyclic reliability indicates that the ICAP-based OECT can sustain repeated synaptic weight updates without performance degradation. Such robust endurance is a key requirement for the practical devices in long-term neuromorphic computing tasks.

Complementarily, we fabricated *n*-type BBL-OECTs using the same ICAP-patterned electrolyte (Fig. 5f). The transfer and output curves (Fig. 5g, h) exhibit accumulation-mode behavior, where I_DS_ increases with positive *V*_GS_. The ICAP-patterned BBL-OECT achieves an on/off ratio exceeding 10^5^, nearly 10 times higher than conventional planar *n*-type OECTs (typically 10^3^ ~ 10^4^), while maintaining an average transconductance of 40 ± 8 μS [66, 67]. Dynamic neuromorphic responses are equally robust. The excitatory postsynaptic current (EPSC) scales with pulse amplitude (Figs. 5i and S17a), and the device achieves ~ 1 ms temporal resolution. Synaptic functionalities, including PPF and LTP, are faithfully reproduced (Fig. S17b, c), and the device retains 85.3% of its initial IDS after 5000 pulsing cycles (Fig. 5j), confirming excellent operational stability. These results together demonstrate that ICAP-patterned electrolytes are universally compatible with both *p*-type and *n*-type organic semiconductors, while enabling high on/off ratios, millisecond-scale synaptic responses, and long-term operational endurance.

**Fig. 6:**
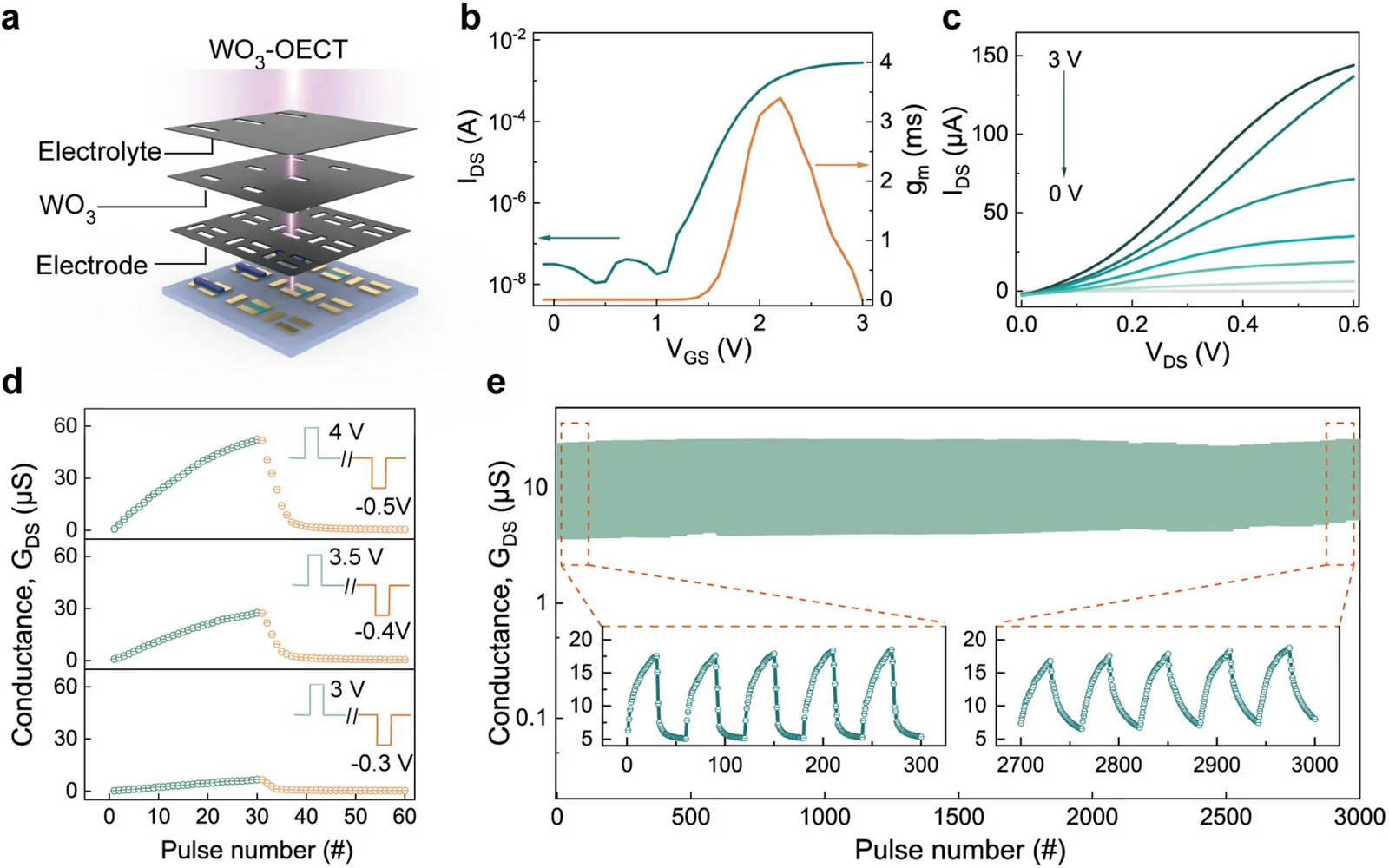
Compatibility of ICAP electrolytes with inorganic semiconductors and system-level neuromorphic applications. **a** Fabrication of WO_3_-OECT. **b**, **c** Transfer and output curves showing on/off ratio of 10^5^. **d** Long-term potentiation (LTP) and depression (LTD) characteristics under varying pulse amplitudes (*V*_*D*_ = 0.5 V, *t*_*P*_ = 0.75 s, *Δt* = 2 s). **e** Endurance of programmable conductance states over 3000 cycles

To further validate the universality, we fabricated a fully lithographically defined OECT using WO_3_ as the channel semiconductor (Fig. 6a). The transfer and output curves (Fig. 6b, c) confirm accumulation-mode operation, with an on/off ratio of 10^5^ and an average transconductance of 3.4 ± 0.6 mS. Importantly, WO_3_ exhibits clear electrochromic modulation, where increasing V_GS_ reduces optical transmittance (Fig. S18). This provides a direct spectroscopic signature of ion-driven doping via the redox transition from W^6+^ to W^5+^ [68], and indicates a direct connection between ionic transport, redox activity, and tunable conductance states in WO_3_ film.

These devices also exhibit robust neuromorphic behavior, including long-term potentiation (LTP) and long-term depression (LTD) under excitatory and inhibitory pulse trains (Figs. 6d and S19). The conductance states of the device scale proportionally with both pulse amplitude and pulse number, enabling precise analog modulation of synaptic weight and making it highly suitable for hardware-based synaptic learning. With + 3/ − 0.3 V pulse trains, programmable conductance states persist over 3000 consecutive stimulation cycles (Fig. 6e). Compared with previously reported oxide-based ECTs (on/off < 10^3^) [69], ICAP-enabled WO_3_-OECT achieves an order-of-magnitude improvement in switching range. Together with the PEDOT:PSS-based *p*-type and BBL-based *n*-type OECTs, the demonstration of WO_3_-OECTs validates the universal compatibility of ICAP-patterned electrolytes across both organic and inorganic semiconductors. Unlike conventional polymer electrolytes that often suffer from poor adhesion or unstable interfaces on oxide surfaces [70], the ICAP electrolyte can be fully lithographically defined without damaging the semiconductor films. This cross-material compatibility lays the foundation for implementing large-scale neuromorphic circuits where reliable and reproducible synaptic behaviors are essential [3, 71, 72].

Finally, the experimentally measured synaptic responses were further used in an MNIST recognition simulation as a preliminary application-level evaluation of possible neuromorphic computing use (Fig. S20). After 50 training epochs, the artificial neural network (ANN) achieved classification accuracies of 99.7% (PEDOT-OECT), 98.97% (WO_3_-OECT), and 90.7% (BBL-OECT) (Fig. S21). These recognition accuracies are comparable to, and in some cases exceed, previously reported OECT-based neuromorphic systems (~ 85%–96%) [3, 73, 74], approaching the performance of leading inorganic memristor benchmarks [75].

We have successfully developed an ICAP-patterned electrolyte that exhibits both exceptional material compatibility and outstanding electrical properties. The electrolyte demonstrates excellent interfacial stability with a range of functional materials, including organic semiconductors (e.g., PEDOT:PSS, BBL) and inorganic oxides (e.g., WO_3_). All transistors fabricated using this electrolyte show high on/off ratios, superior transconductance, and excellent long-term stability. These characteristics are not only comparable to but often surpass those of previously reported devices (Table S5). Looking forward, given its material versatility and stable device performance, the ICAP process is expected to ensure compatibility and integration with CMOS circuits, facilitating the development of more advanced and intelligent neuromorphic architectures beyond handwritten digit recognition, including temporal sequence learning and multimodal sensory fusion [76, 77].

## Conclusions

In conclusion, we developed an ion compensation-assisted photolithography strategy for high-resolution patterning of solid electrolytes in OECTs. By combining photopatterning with post-patterning ion replenishment, ICAP overcomes a long-standing trade-off between electrolyte patternability and ionic functionality, enabling finely patterned structures retaining high capacitance and operational stability. This capability supports reliable transistor operation, reduced parasitic coupling, and scalable array integration across multiple semiconductor materials. Beyond device-level operation, ICAP also enables neuromorphic functions, underscoring its broader applicability in complex circuits. Overall, ICAP provides a versatile and practical framework for high-density electrolyte-gated electronics, and opens new opportunities for integrated neuromorphic, flexible, and bioelectronic systems.

## Supplementary Information

Below is the link to the electronic supplementary material.

Supplementary file1 (DOCX 5218 KB)
