# Supplementary material for: Ion Compensation-Assisted Photolithography Enables High-Resolution Electrolytes for Neuromorphic Transistors
Source: Nanomicro Lett. 2026 Jul 13;18:434. doi: 10.1007/s40820-026-02288-4 (PMC13357476; doi:10.1007/s40820-026-02288-4)
Supplement: Supplementary file 1 — Supplementary file1 (DOCX 5218 KB) [file 40820_2026_2288_MOESM1_ESM.docx]

Supporting Information for

**Ion Compensation-Assisted Photolithography Enables High-Resolution Electrolytes for Neuromorphic Transistors**

Wenjing Zhang^1^, Xiaoci Liang^1^, Sixing Chen^1^, Xiuquan Ma^4^, Chen Chen^3,^ *, Songjia Han^2,^ *, and Chuan Liu^1,^ *

^1^ State Key Laboratory of Optoelectronic Materials and Technologies and Guangdong Province Key Laboratory of Display Material and Technology, School of Electronics and Information Technology, Sun Yat-Sen University, Guangzhou 510275, P. R. China

^2^ College of Artificial Intelligence and Low-Altitude Technology, South China Agricultural University, Guangzhou 510642, P. R. China

^3^ Science and Technology on Advanced Ceramic Fibers and Composites Laboratory, College of Aerospace Science and Engineering, National University of Defense Technology, Changsha 410073, P. R. China

^4^ Guangdong HUST Industrial Technology Research Institute, Guangdong Provincial Key Laboratory of Digital Manufacturing Equipment, Dongguan 523000, P. R. China

* Corresponding authors. E-mail: liuchuan5@mail.sysu.edu.cn (Chuan Liu); hansongjia@scau.edu.cn (Songjia Han); angela_chen@nudt.edu.cn (Chen Chen)

**Supporting Figures**


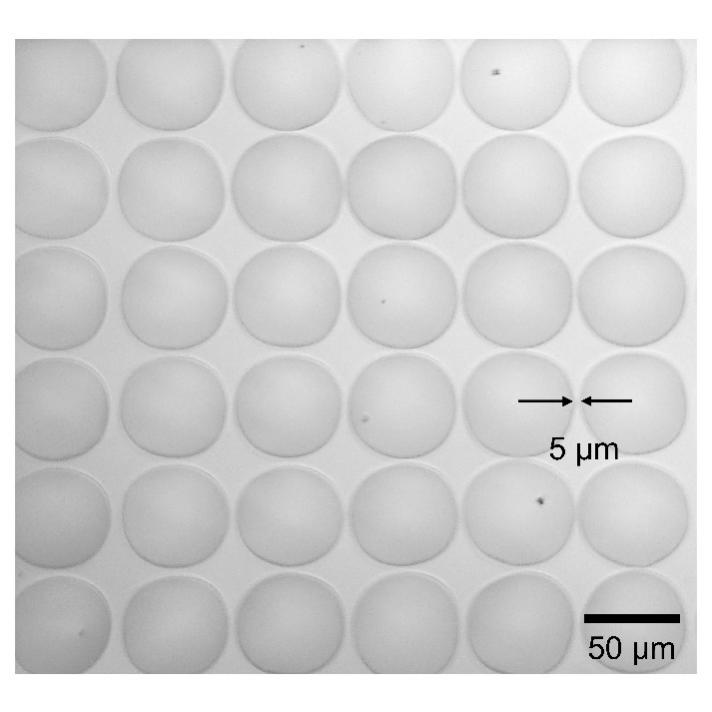


**Fig. S1** Optical microscopy images of the PLE with the resolution interval down to 5 μm. Note the minimum feature of 2 μm is shown in the main text (Fig. 2a)

**
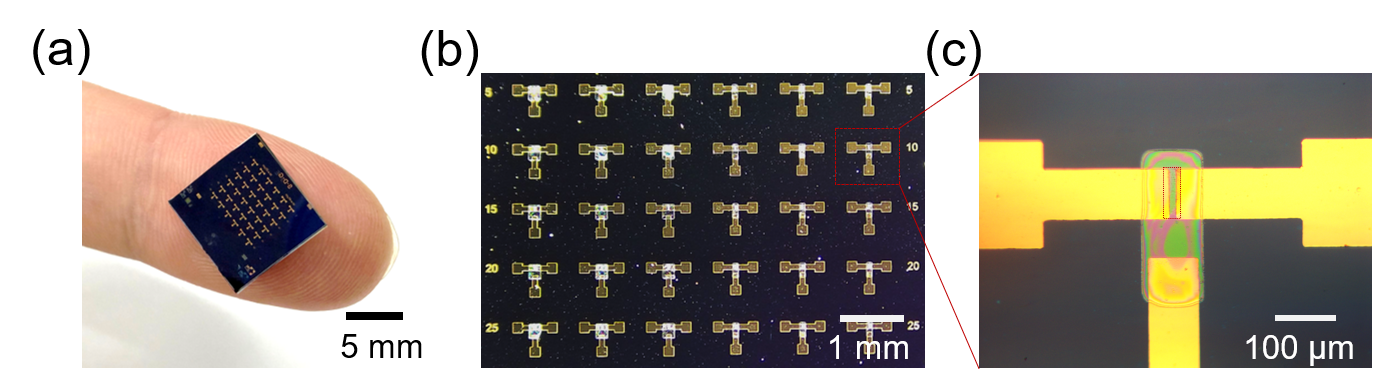
**

**Fig. S2** Array-level demonstration of ICAP-patterned OECTs with various channel lengths (e.g., 5 μm, 10 μm, 15 μm, 20 μm, 25 μm): (a) Photograph of the photolithographically patterned WO_3_-OECT array; (b) Optical image of the WO_3_-OECT array; (c) Enlarged optical microscopy image of an individual WO_3_-OECT, with the dashed region indicating the photolithographically patterned WO_3_


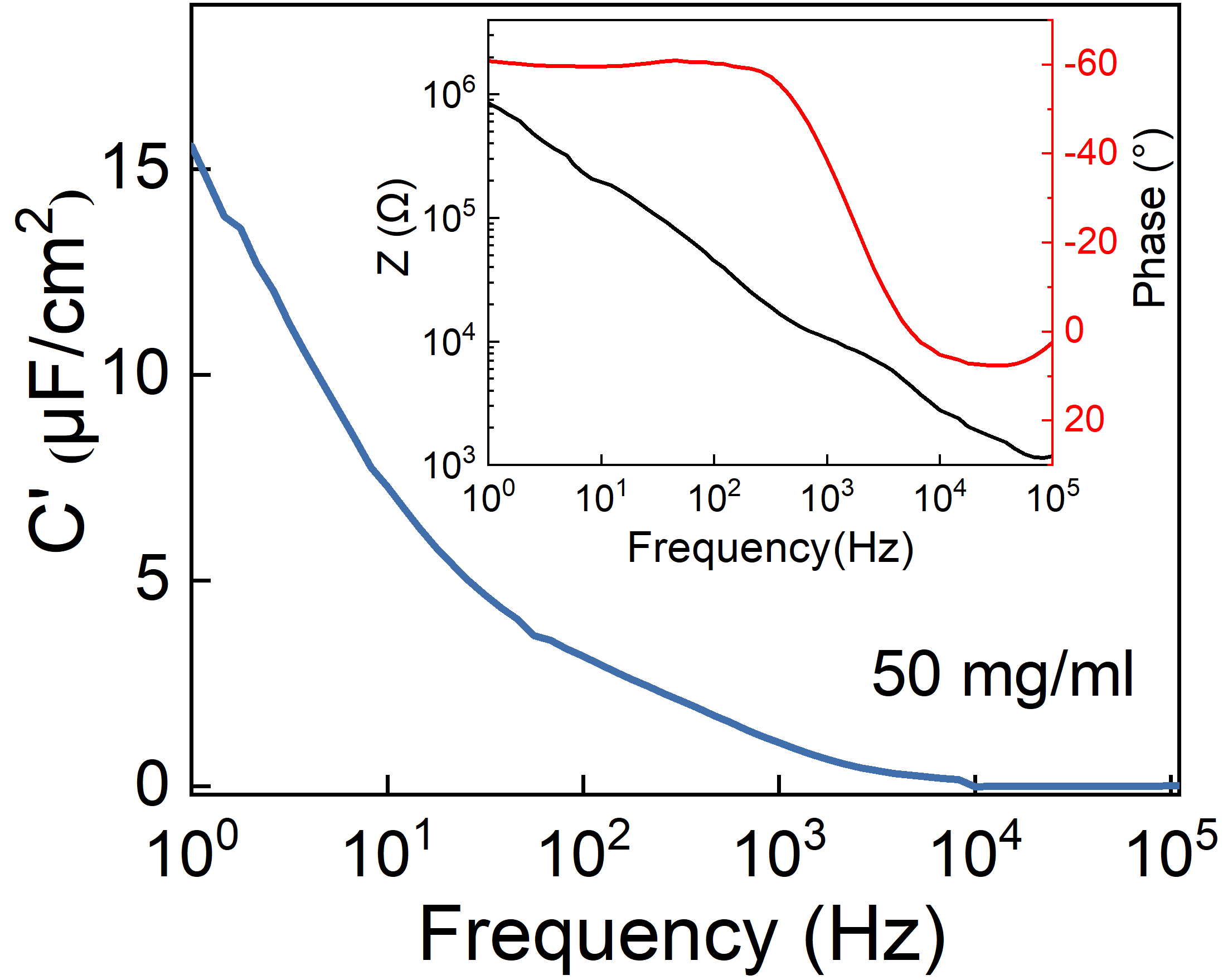


**Fig. S3** Frequency-dependent capacitance of the PLE in 50 mg/mL LiCl solution


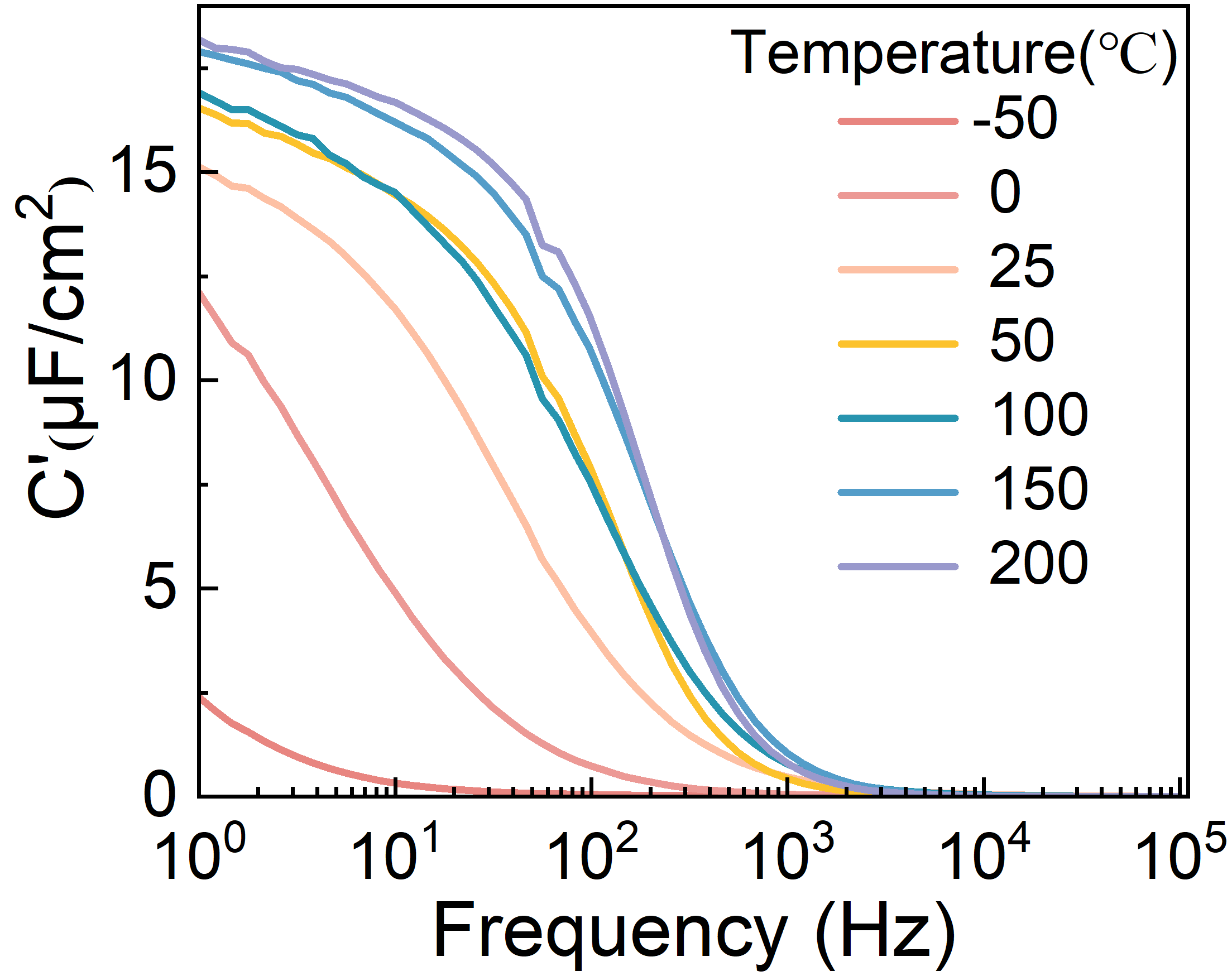


**Fig. S4** Capacitance-frequency response of the PLE at different temperatures

**
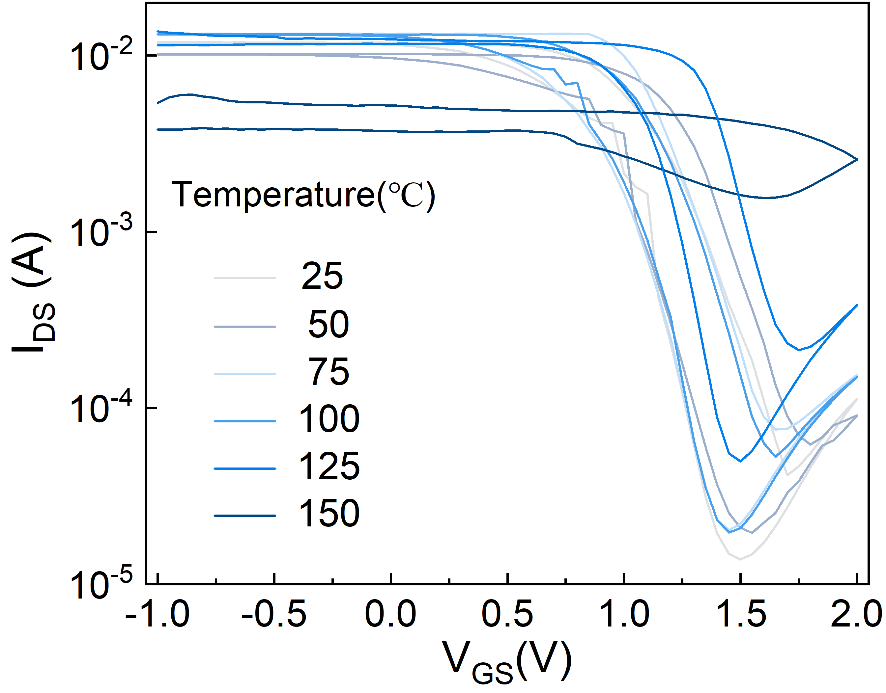
**

**Fig. S5** Thermal validation of PEDOT:PSS-OECTs with ICAP-patterned electrolyte


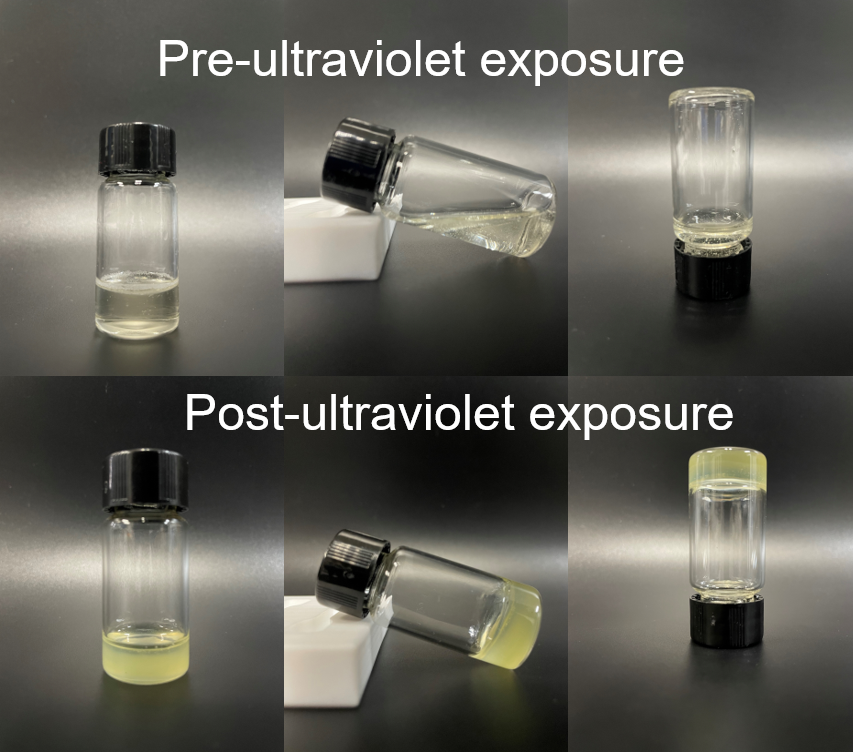


**Fig. S6** Images of the PLE sol-gel transition under ultraviolet radiation

**
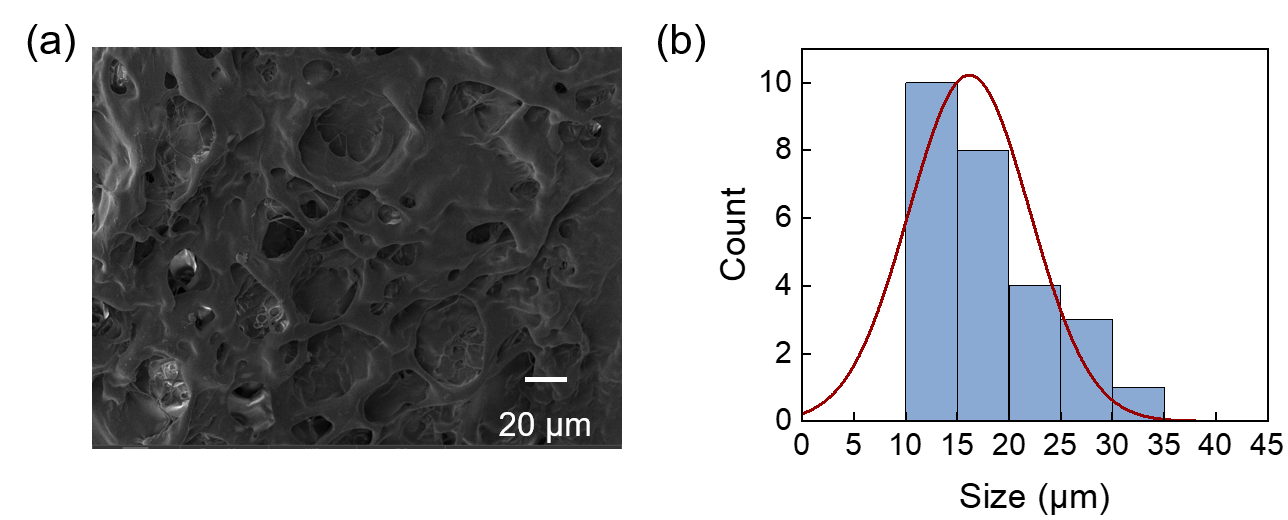
**

**Fig. S7** Surface morphology of the HPC-gel framework: (a) Representative SEM image of HPC-gel; (b) Statistical analysis of pore/free-volume features extracted from SEM images

**Fig. S8** XPS O1s spectrum of HPC and PLE


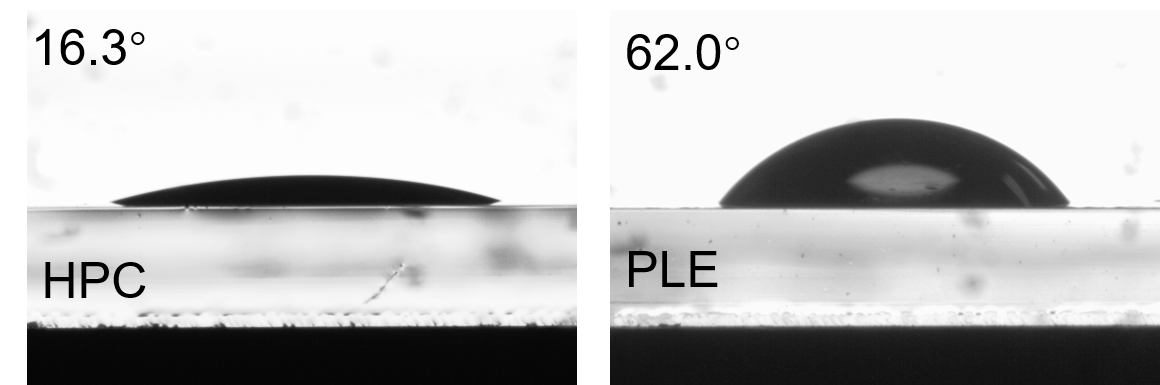


**Fig. S9** Comparison of contact angles for HPC and PLE

**
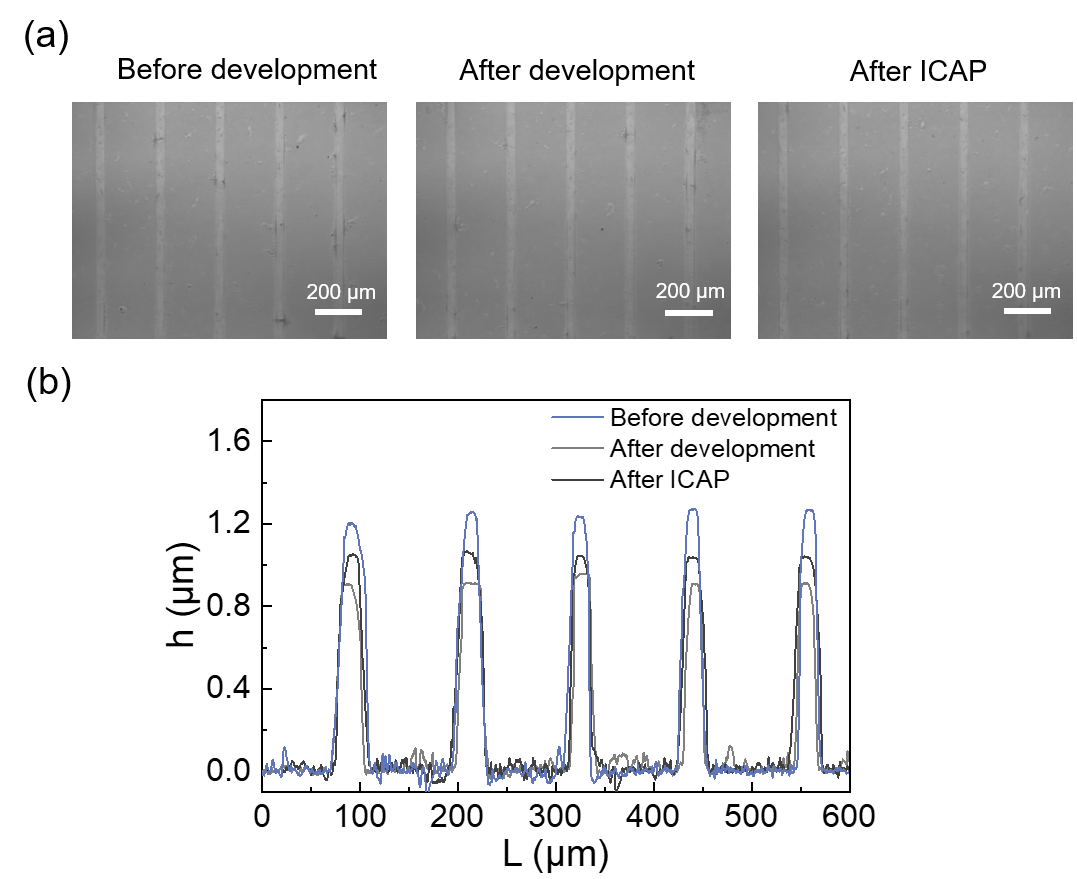
**

**Fig. S10** Stability of patterned ICAP electrolyte during development and ion compensation. (a) Optical images of line-patterned electrolyte arrays before development, after development, and after ICAP treatment; (b) Profilometer height profiles of the line-patterned electrolyte arrays

**
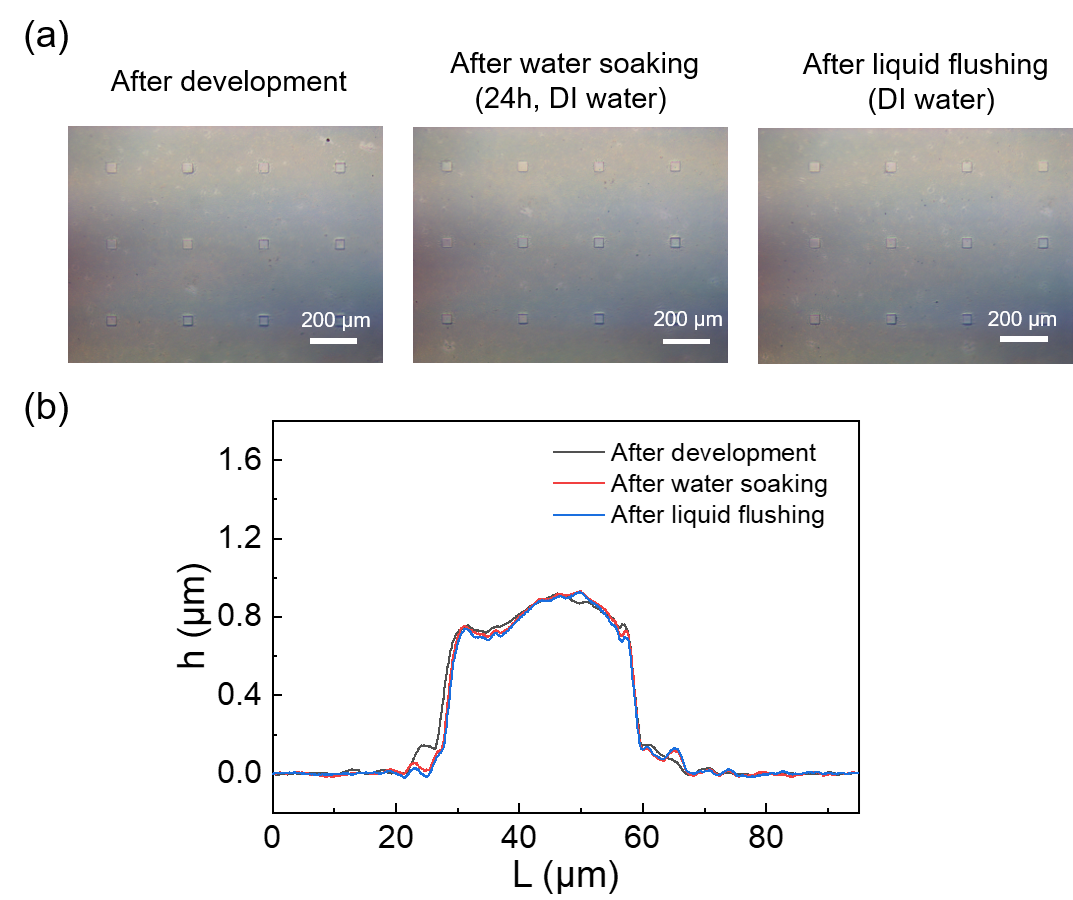
**

**Fig. S11** Stability of the patterned electrolyte under prolonged water immersion: (a) Optical images of patterned electrolyte after development, after 24 h soaking in DI water, and after DI-water flushing; (b) Profilometer height/thickness profiles of a single electrolyte pattern after development, after 24 h DI-water soaking, and after DI-water flushing


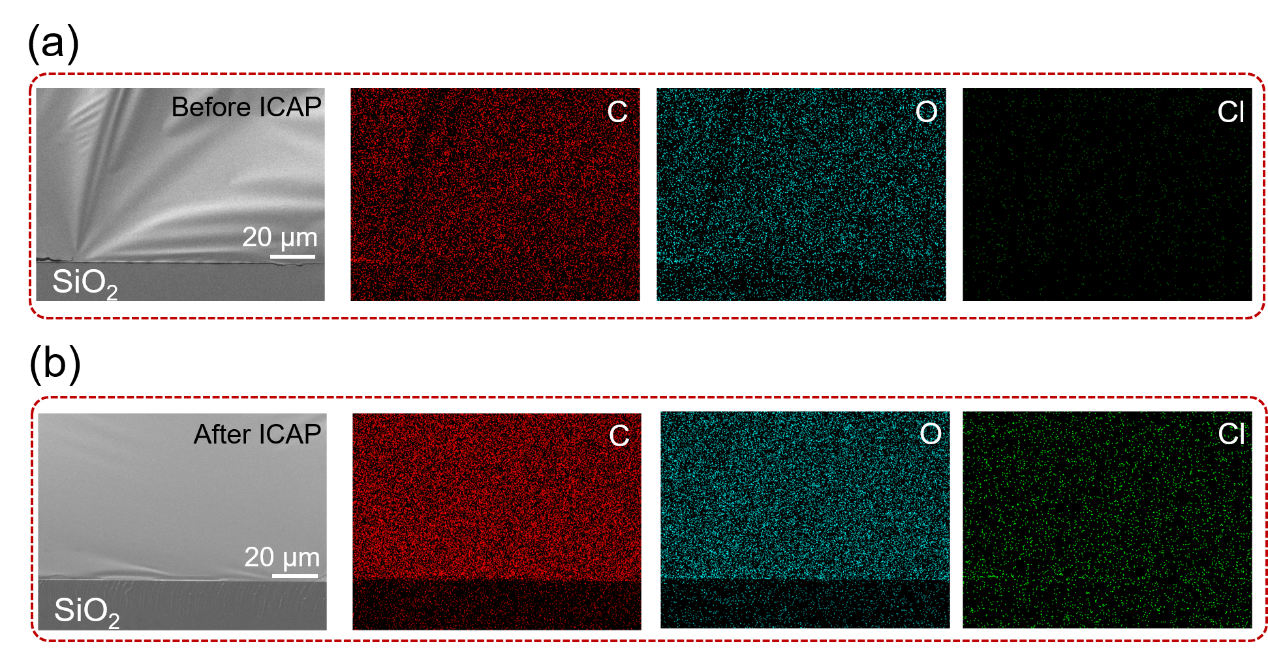


**Fig. S12** Cryo-fractured cross-sectional SEM images and corresponding C, O, and Cl elemental mapping of ICAP electrolyte films on SiO_2_ substrates before (a) and after (b) ICAP treatment


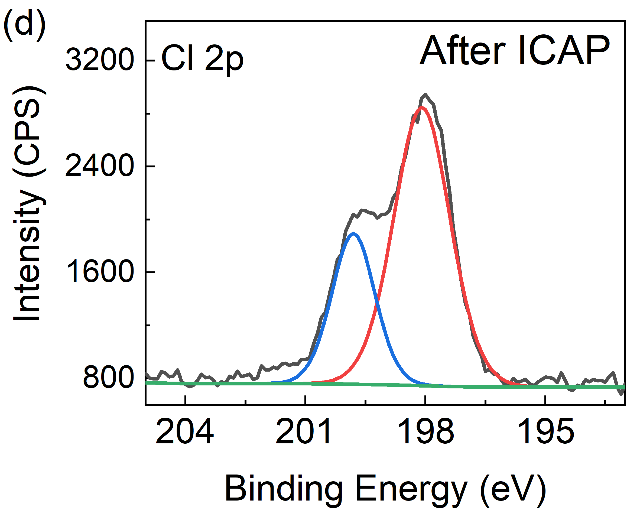

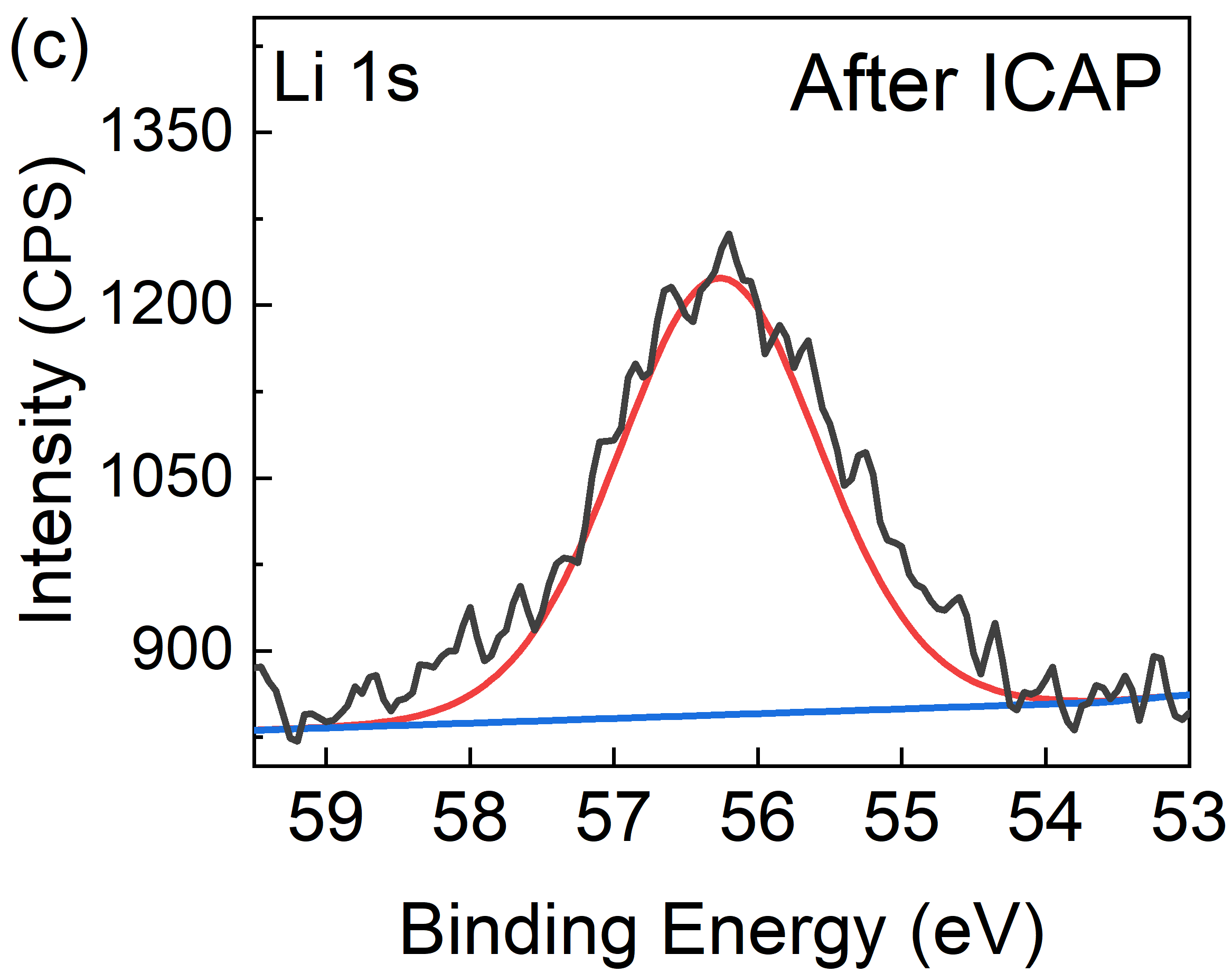

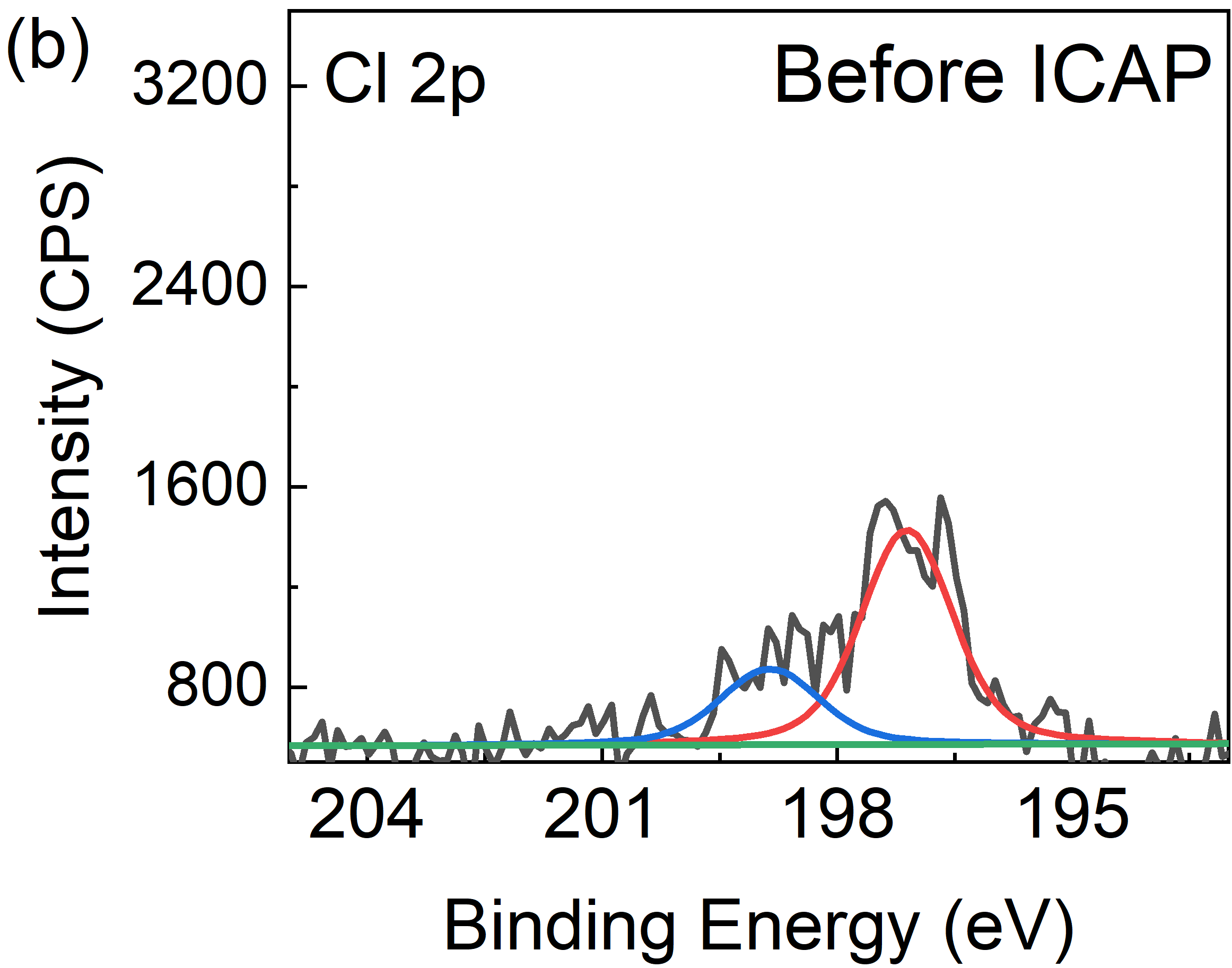

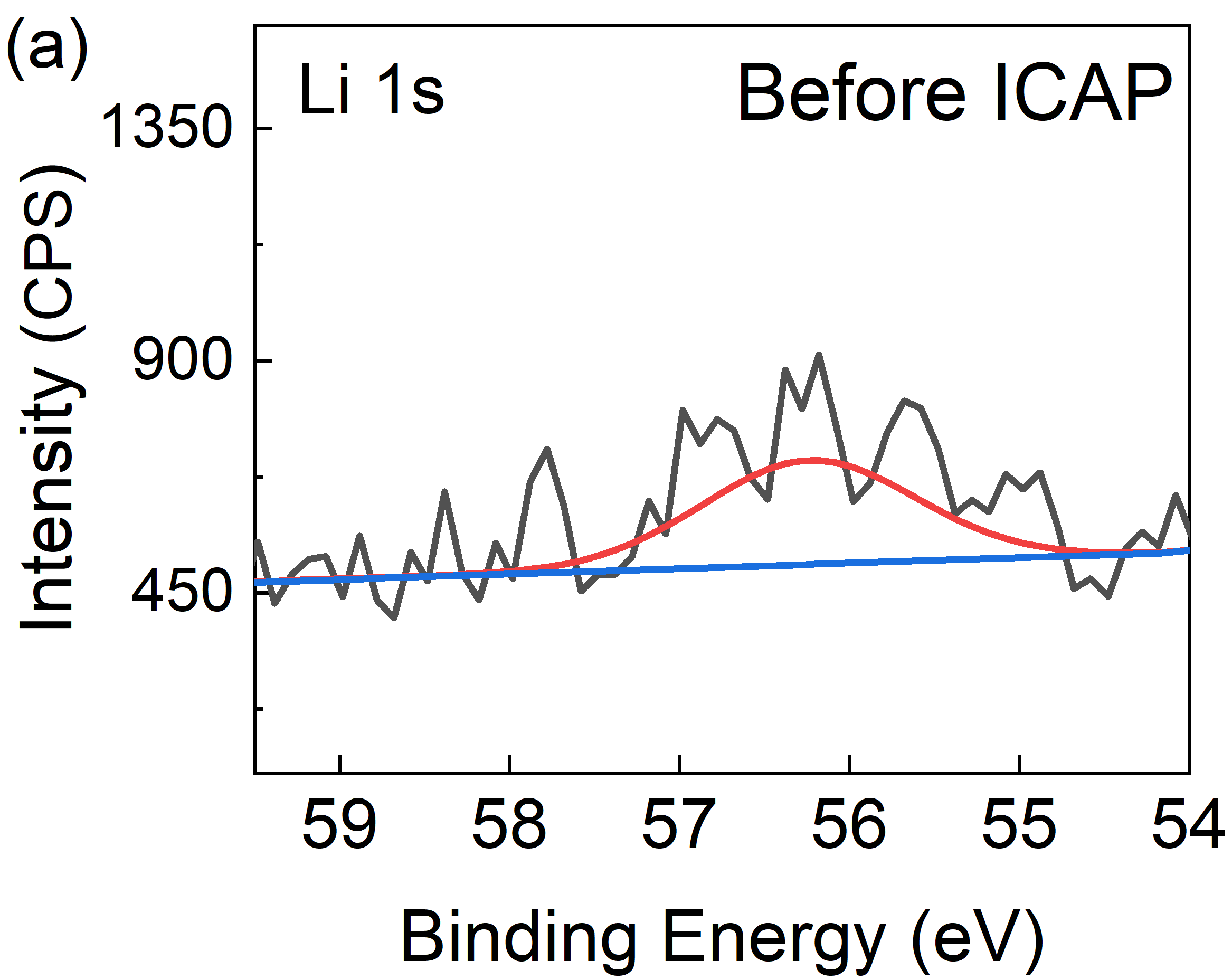


**Fig. S13** XPS Li 1s and Cl 2p spectra of the PLE before and after immersion in LiCl solution


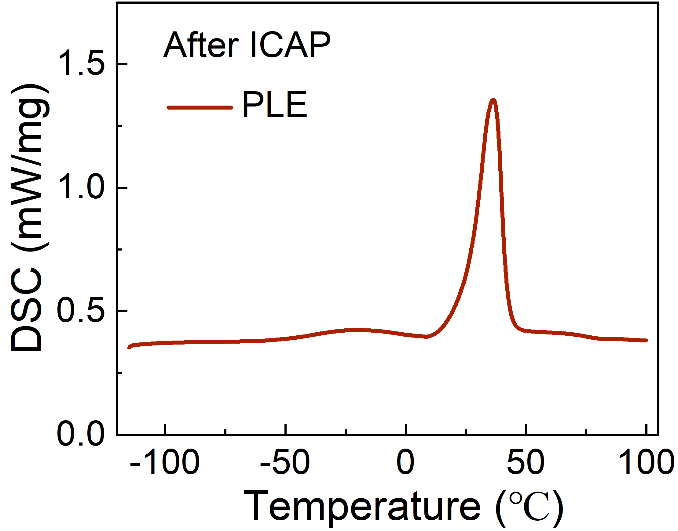


**Fig. S14** Differential scanning calorimetry (DSC) thermogram of the PLE


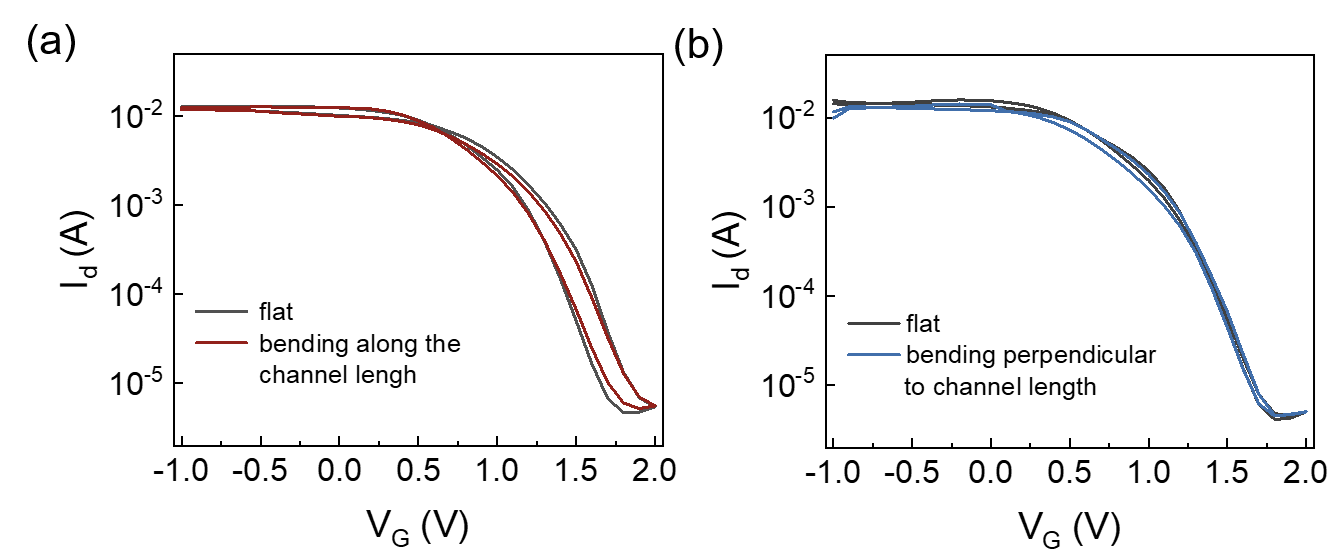


**Fig. S15** Transfer curves of PEDOT-OECTs measured in the initial flat state and after 200 bending cycles: (a) bending parallel to the channel-length. (b) bending perpendicular to the channel-length


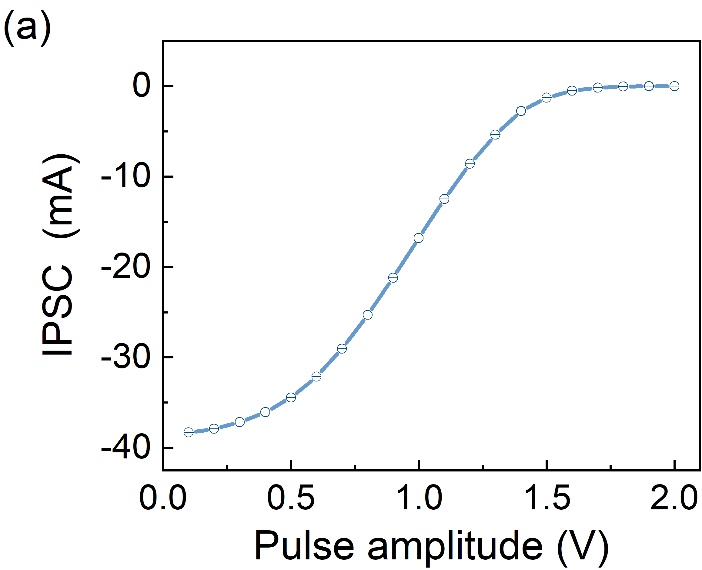


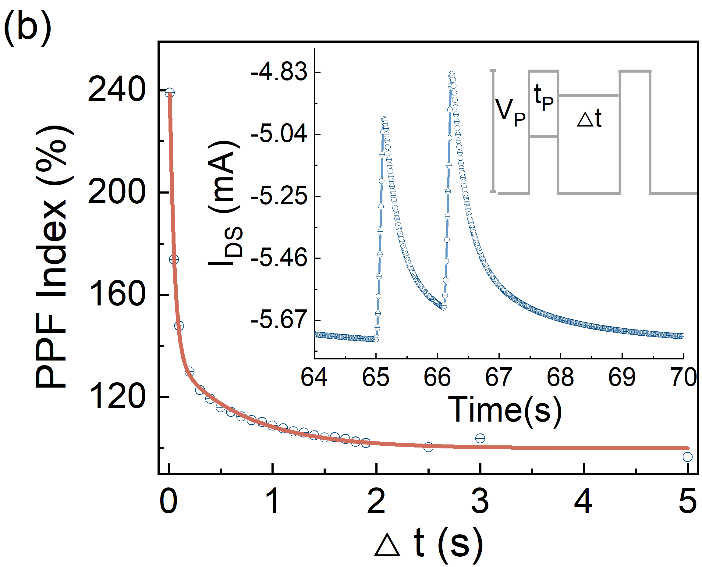


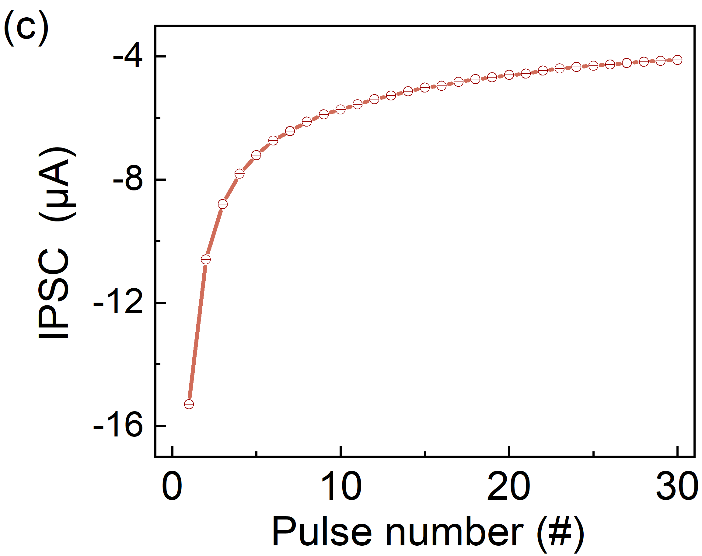


**Fig. S16** (a) IPSC response of PEDOT-OECT under voltage stimulation with varying intensities. (b) Paired-pulse facilitation (PPF) effect and synaptic weight change at V_D_=−0.6V, V_P_=1.5V, and t_P_=100 ms. (c) Long-term plasticity (LTP) under 50 ms repetitive stimulations at V_D_=−0.6V, V_P_=1V, and Δt=150 ms


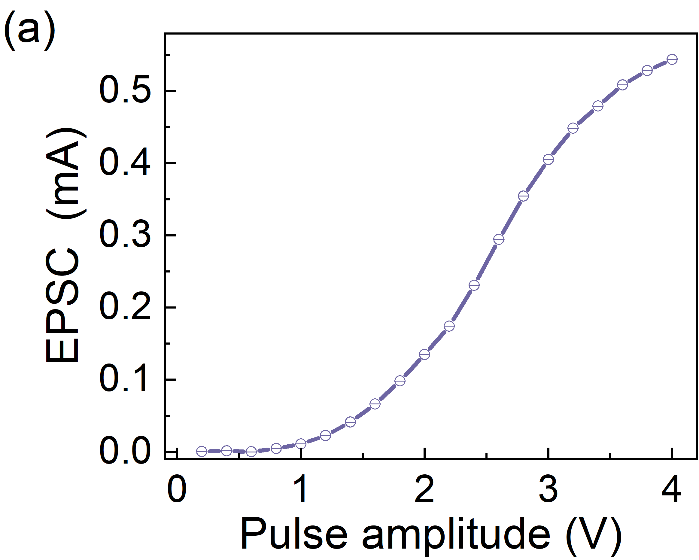

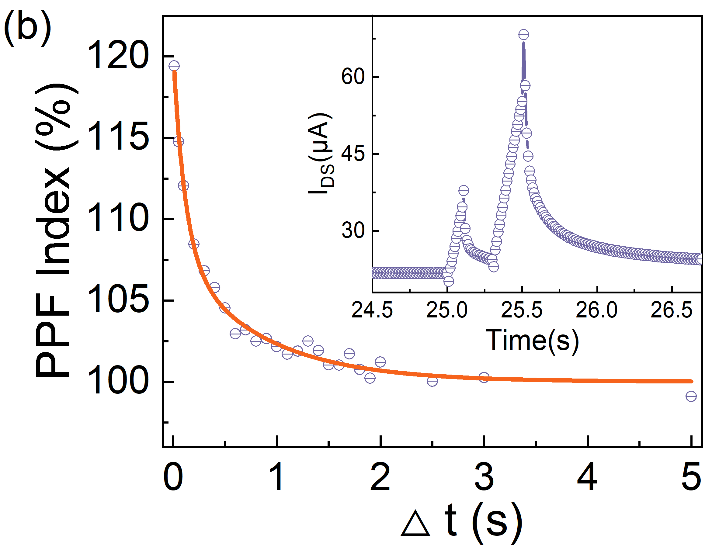

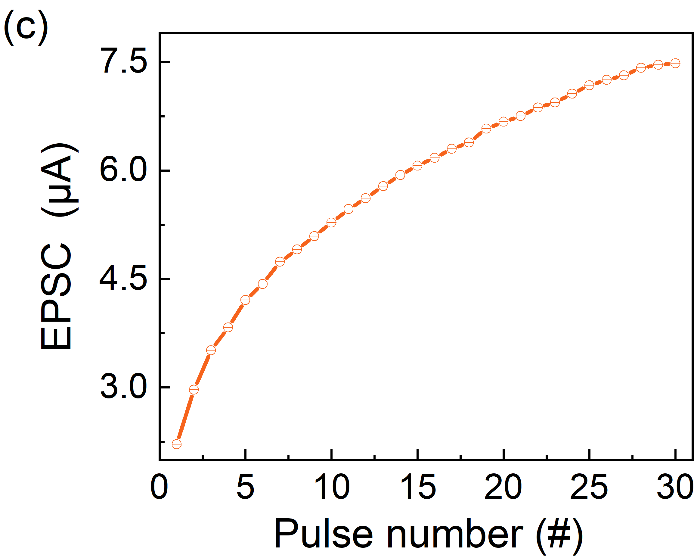


**Fig. S17** (a) EPSC response of BBL-OECT under voltage stimulation with various pulse intensities. (b) PPF and synaptic weight change at V_D_=0.6V, V_P_=2V, and t_P_=100 ms. (c) LTP under 50 ms repetitive stimulation at V_D_=0.6V, V_P_=2V, and Δt=100 ms

**Fig. S18** Transmission spectra of WO_3_-OECT at various gate voltages


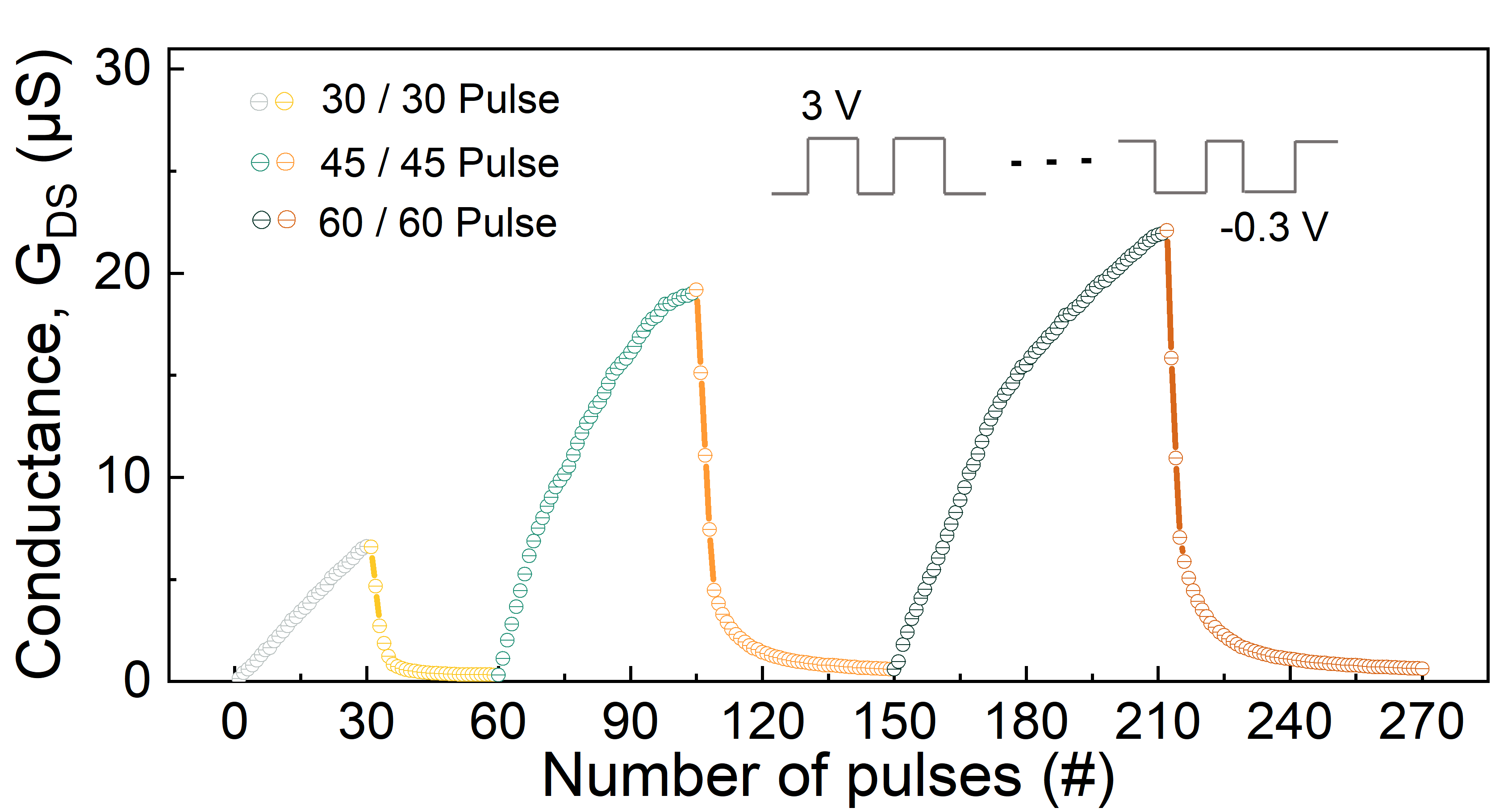


**Fig. S19** Conductance response of WO_3_-OECT at various pulse numbers (V_D_ = 0.5 V, t_P_ = 0.75 s, Δt = 2 s)

**
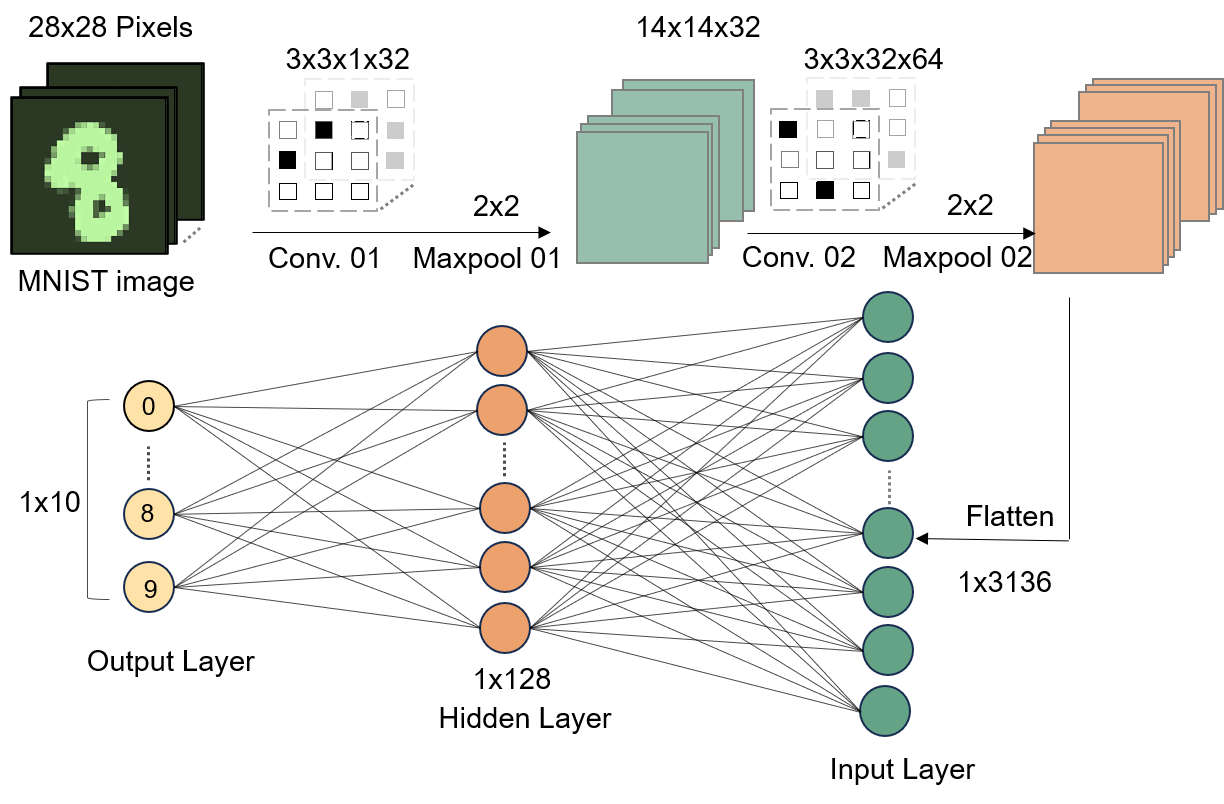
**

**Fig. S20** Schematic of ANN simulations with device conductance mapped as synaptic weights

**
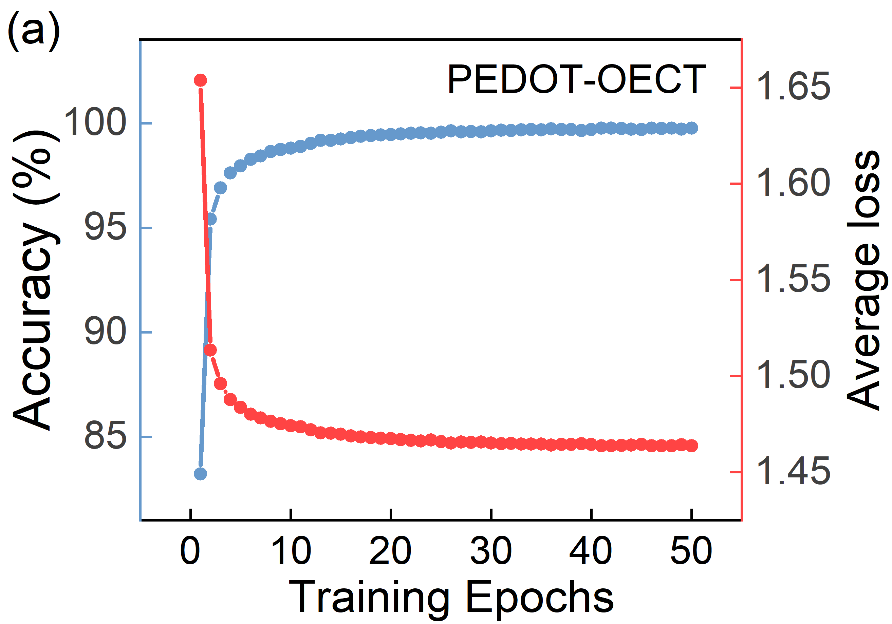
**
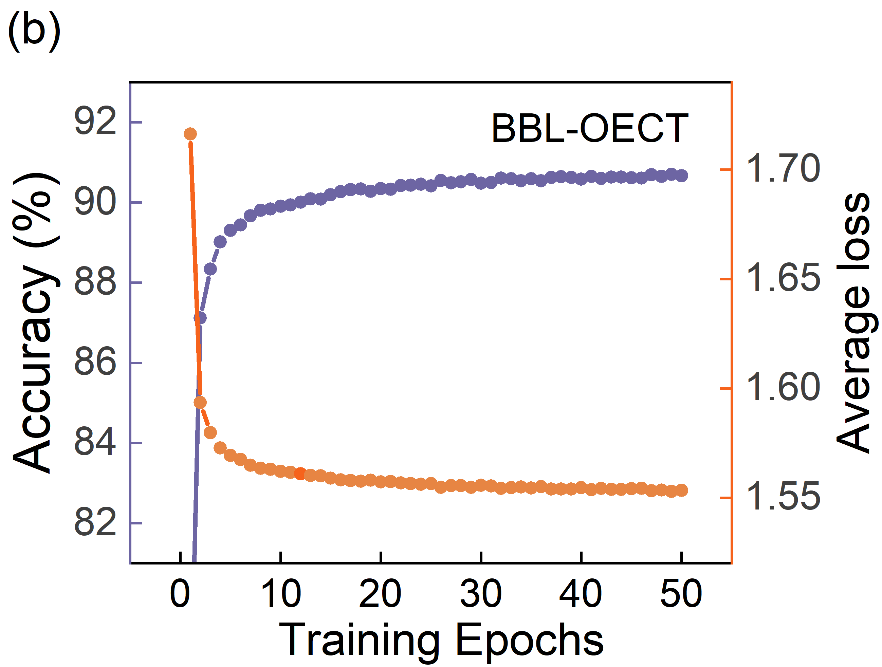
**
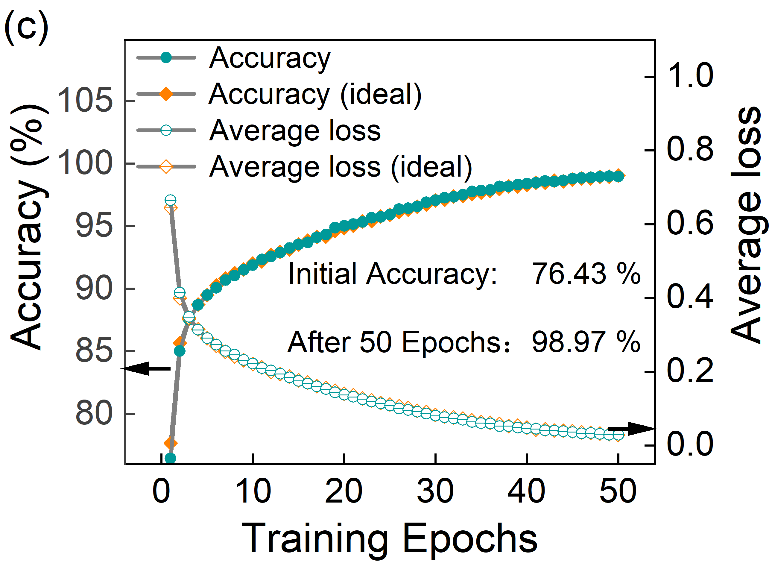
**

**Fig. S21** (a) Recognition accuracy and average loss for the PEDOT-OECT over 50 training epochs on hand-written digits (MNIST). (b) Recognition accuracy and average loss for the BBL-OECT over 50 training epochs on MNIST. (c) Recognition accuracy and average loss for the WO_3_-OECT over 50 training epochs on MNIST

**Supplementary Tables**

**Table S1** Comparison of patterning resolution, capacitance, and ionic conductivity of ICAP-fabricated electrolytes with reported electrolytes

| Year | Material | Pattern Size | Technique | Capacitance  (μF/cm^2^) | Conductivity  (mS/cm) | Ref. |
| --- | --- | --- | --- | --- | --- | --- |
| 2018 | PDA | 100 μm | electrospinning | N/A | 2 | [S1] |
| 2018 | PEGDA | 50 μm | photolithography | N/A | 0.047 | [S2] |
| 2019 | PVDF-HFP | 40 μm | printing | 5 | 1.1 | [S3] |
| 2020 | PMMA | 5 μm | tape-casting | N/A | N/A | [S4] |
| 2020 | PTFE | 0.5 mm | inkjet printing | N/A | N/A | [S5] |
| 2021 | Nafion | 50 μm | imprint lithography | N/A | N/A | [S6] |
| 2022 | 8YSZ | 125 μm | mesh pressing | N/A | N/A | [S7] |
| 2022 | BCZYYb | 39.5 μm | soft lithography | 0.003 | 5 | [S8] |
| 2022 | Nafion/PDMS | 5 μm | soft lithography | N/A | 43.06 | [S9] |
| 2022 | NIPAM | 10 μm | photolithography | N/A | 24 | [S10] |
| 2023 | PAN | 16 μm | electrospinning | N/A | 0.367 | [S11] |
| 2023 | PVA/PAAM | 400 μm | photolithography | 1.6 | 0.03 | [S12] |
| 2023 | 8YSZ | 100 μm | 3D printing | N/A | 36.6 | [S13] |
| 2024 | HACC | 20 μm | photolithography | 1.6 | 4×10^-4^ | [S14] |
| 2025 | PEGDMA | 15 μm | photolithography | N/A | 10 | [S15] |
| 2025 | CS-PHEA | 300–400 μm | photolithography | N/A | 0.258 | [S16] |
| 2025 | pDADMAC | 25 μm | photolithography | N/A | N/A | [S17] |
| 2025 | NBR/LiTFSI | 200 μm | photolithography | 10 | 1.95×10^-4^ | [S18] |
| 2026 | SU-8 | 25 μm | photolithography | N/A | 17.1 | [S19] |
| 2026 | PVA/PAAm | 66.2 μm | inkjet printing | N/A | N/A | [S20] |
| 2026 | HPC | 2 μm | photolithography | 15.6 | 6.7 | This work |

**Table S2** Semi-quantitative EDS elemental composition of electrolyte film surfaces before and after ICAP treatment

| Sample | C  Atomic % | O  Atomic % | Cl  Atomic % | C  Weight % | O  Weight % | Cl  Weight % |
| --- | --- | --- | --- | --- | --- | --- |
| Before ICAP | 58.06 | 41.87 | 0.08 | 50.90 | 48.90 | 0.20 |
| After  ICAP | 56.85 | 38.27 | 4.89 | 46.50 | 41.70 | 11.80 |

**Table S3** Semi-quantitative EDS elemental composition of cryo-fractured cross-sections of electrolyte films before and after ICAP treatment

| Sample | C  Atomic % | O  Atomic % | Cl  Atomic % | C  Weight % | O  Weight % | Cl  Weight % |
| --- | --- | --- | --- | --- | --- | --- |
| Before ICAP | 2.08 | 97.92 | 0.00 | 1.57 | 98.43 | 0.00 |
| After  ICAP | 2.99 | 90.79 | 6.22 | 2.10 | 84.99 | 12.91 |

**Table S4** The parameters used in the simulations of a transistor

| **Symbol** | **Value** | **Unit** | **Description** |
| --- | --- | --- | --- |
| *t*_elec_ | 100 | nm | Thickness of the electrolyte layer |
| *t*_semi_ | 70 | nm | Thickness of the semiconductor layer |
| *t*_ox_ | 100 | nm | Thickness of the insulating layer |
| *W* | 400 | μm | Width of the channel |
| *L* | 1-10 | μm | Length of the channel |
| *c*_+0_ | 10^20^ | cm^-3^ | Initial cation concentration in electrolyte |
| *c*_-0_ | 10^20^ | cm^-3^ | Initial anion concentration in electrolyte |
| $D_{\mathrm{ie}}$ | 6.67e-6 | cm^2^/s | Diffusion coefficient in electrolyte layer |
| $D_{\mathrm{is}}$ | 6.67e-8 | cm^2^/s | Diffusion coefficient in semiconductor layer |
| *ε*_e_ | 3.9 | / | Relative dielectric constant of the electrolyte layer |
| *ε*_s_ | 10 | / | Relative dielectric constant of the semiconductor layer |
| *E*_g_ | 3.7 | eV | The bandgap of the semiconductor |
| *W*_F_ | 4.17 | eV | Workfunction of source/drain electrodes |
| *φ*_s_ | 4.16 | eV | Affinity of the semiconductor |
| *μ*_semi_ | 15 | cm^2^/Vs | Mobility of the semiconductor |

**Table S5** Compared with reported electrolyte-gated transistor in prior studies

| Channel | Material  type | Electrolyte | on/off ratio | g_m_  (mS) | μC*  (F cm^-1^V^-1^s^-1^) | Stability | Ref. |
| --- | --- | --- | --- | --- | --- | --- | --- |
| PEDOT:PSS | p | 0.1 M NaCl | 10^2^ | 20.2 | N/A | N/A | [S21] |
| PEDOT:PSS | p | PVA-hydrogel | 1.5 × 10^3^ | 54 | 91 ± 19 | 1000 | [S22] |
| PEDOT:PSS | p | LiClO_4_ | 0.3 × 10^3^ | 0.416 | 0.35 | N/A | [S23] |
| g2T-T | p | 0.1 M NaCl | 10^4^ | 12.29 | 288.9 | 1000 | [S24] |
| g2T-S | p |  | 10^4^ | 14.45 | 332.7 | 1000 |  |
| g2T-SVS | p |  | 10^5^ | 10.33 | 285.0 | 1000 |  |
| pgBTTT | p | E300-RISE | 1.6 × 10^4^ | 0.32 | 15 | 7200 | [S25] |
| p(g3C2T2-T) | p | PBS | 5.4 × 10^3^ | 29.7 | 800 | 360 | [S26] |
| BBL-H | n | 0.1 M KCl | 1.59 × 10^4^ | 10.8 | 9.27 ± 0.03 | 100 | [S27] |
| BBL-L | n |  | 1.23 × 10^4^ | 0.41 | 0.28 ± 0.01 | 100 |  |
| Cl_2_-BAL | n | 0.1 M NaCl | 10^5^ | 0.45 | 6.20 ± 1.25 | 50000 | [S28] |
| f-BTI2g-TVTF | n | 0.1 M NaCl | 10^5^ | 1.062 | 90.2 ± 10.5 | 100 | [S29] |
| PDPPODT-TT | n | AuCl_3_ | 2.34 × 10^5^ | 1.51 × 10^-2^ | 0.21 | N/A | [S30] |
| gNDI-EDBT | n | 0.1 M NaCl | 10^3^ | 5.2 × 10^-2^ | 0.02 ± 0.01 | 360 | [S31] |
| gNDI-BT | n |  | 10^4^ | 0.3 | 0.09 ± 0.01 | 360 |  |
| gNDI-FBT | n |  | 10^4^ | 0.8 | 0.12 ± 0.02 | 360 |  |
| p(NDI-T2-L2) | n | 0.1 M NaCl | 2.2 × 10^2^ | 2.4 × 10^-3^ | 0.0023 | 100 | [S32] |
| BBL | n | 0.1 M NaCl | 1.6 × 10^3^ | 6.51 × 10^-2^ | 1.99 | N/A | [S33] |
| BBL_15_ | n | 0.1 M NaCl | 2.9 × 10^3^ | 2.47 × 10^-3^ | 1.94 ± 0.05 | 1000 | [S34] |
| BBL_60_ | n |  | 8.3 × 10^4^ | 7.68 × 10^-3^ | 4.90 ± 0.16 | 1000 |  |
| BBL_98_ | n |  | 2.0 × 10^5^ | 1.62 × 10^-2^ | 10.2 ± 0.4 | 1000 |  |
| BBL_152_ | n |  | 4.4 × 10^5^ | 4.44 × 10^-2^ | 25.9 ± 0.9 | 1000 |  |
| Li_x_TiO_2_ | Metal oxide | LiClO_4_/PEO | 1.7 | 7.5 × 10^-2^ | 66.7 | 1000 | [S35] |
| LiCoO_2_ | Metal oxide | Li3PO_x_Se_x_ | 19.3 | 4.06 × 10^-5^ | 0.02 | 720 | [S36] |
| W-InO_x_ | Metal oxide | AlO_x_ | 10^4^ | N/A | N/A | N/A | [S37] |
| In_2_O_3_ | Metal oxide | SiO_2_ | 10^3^ | 1.77 | N/A | N/A | [S38] |
| WO_3_ | Metal oxide | HfO_x_ | 679 | 5.8 × 10^-4^ | 4.5 | 1000 | [S39] |
| WO_3-x_ | Metal oxide | HfO_2_ | 112 | 5.6 × 10^-3^ | 3.8 | 2000 | [S40] |
| WO_3_ | Metal oxide | ZrO_2_ | 20 | N/A | N/A | 10^7^ | [S41] |
| WO_3_ | Metal oxide | Nafion | 10^5^ | N/A | N/A | N/A | [S42] |
| PEDOT:PSS | p | ICAP-electrolyte | 10^4^ | 16.5 | 23.8 | 10000 | **This work** |
| BBL | n |  | 10^5^ | 4 × 10^-2^ | 0.282 | 5000 |  |
| WO_3_ | Metal oxide |  | 10^5^ | 3.4 | 60.7 | 3000 |  |

**Supplementary References**

1. G.H. Kim, H. Nam, W. Choi, T. An, G. Lim, Electrospinning nanofiber on an insulating surface with a patterned functional electrolyte electrode. Adv. Mater. Interfaces **5**(5), 1701204 (2018). <https://doi.org/10.1002/admi.201701204>
2. Y. Zhong, G.T.M. Nguyen, C. Plesse, F. Vidal, E.W.H. Jager, Highly conductive, photolithographically patternable ionogels for flexible and stretchable electrochemical devices. ACS Appl. Mater. Interfaces **10**(25), 21601–21611 (2018). <https://doi.org/10.1021/acsami.8b03537>
3. J.H. Lee, Y.S. Park, S. Cho, I.S. Kang, J.K. Kim et al., Output voltage modulation in triboelectric nanogenerator by printed ion gel capacitors. Nano Energy **54**, 367–374 (2018). <https://doi.org/10.1016/j.nanoen.2018.10.016>
4. C. Lee, S.S. Shin, J. Choi, J. Kim, J.-W. Son et al., A micro-patterned electrode/electrolyte interface fabricated by soft-lithography for facile oxygen reduction in solid oxide fuel cells. J. Mater. Chem. A **8**(32), 16534–16541 (2020). <https://doi.org/10.1039/D0TA03997G>
5. I. Bae, B. Kim, D.-Y. Kim, H. Kim, K.-H. Oh, In-plane 2-D patterning of microporous layer by inkjet printing for water management of polymer electrolyte fuel cell. Renew. Energy **146**, 960–967 (2020). <https://doi.org/10.1016/j.renene.2019.07.003>
6. R. Umezaki, J. Murata, Electrochemical imprint lithography on Si surface using a patterned polymer electrolyte membrane. Mater. Chem. Phys. **259**, 124081 (2021). <https://doi.org/10.1016/j.matchemphys.2020.124081>
7. C. Timurkutluk, T. Altan, S. Onbilgin, F. Yildirim, M. Yagiz et al., Mesh patterned electrolyte supports for high-performance solid oxide fuel cells. Int. J. Energy Res. **46**(8), 10689–10703 (2022). <https://doi.org/10.1002/er.7872>
8. C. Lee, S.S. Shin, J. Kim, J. Choi, M. Choi et al., Tailoring an interface microstructure for high-performance reversible protonic ceramic electrochemical cells *via* soft lithography. ACS Appl. Mater. Interfaces **14**(28), 32124–32133 (2022). <https://doi.org/10.1021/acsami.2c08918>
9. H. Lee, H. Seo, S.K. Kim, I. Bae, Aligned proton transport highway of hierarchically structured proton-exchange membranes constructed *via* capillary force lithography. ACS Appl. Energy Mater. **5**(5), 6256–6264 (2022). <https://doi.org/10.1021/acsaem.2c00622>
10. A. Weissbach, L.M. Bongartz, M. Cucchi, H. Tseng, K. Leo et al., Photopatternable solid electrolyte for integrable organic electrochemical transistors: operation and hysteresis. J. Mater. Chem. C **10**(7), 2656–2662 (2022). <https://doi.org/10.1039/d1tc04230k>
11. Q. Kang, Z. Zhuang, Y. Liu, Z. Liu, Y. Li et al., Engineering the structural uniformity of gel polymer electrolytes *via* pattern-guided alignment for durable, safe solid-state lithium metal batteries. Adv. Mater. **35**(38), 2303460 (2023). <https://doi.org/10.1002/adma.202303460>
12. J. Kang, K.Y. Kim, S. Kim, H. Hong, B.-S. Bae et al., A conformable microneedle sensor with photopatternable skin adhesive and gel electrolyte for continuous glucose monitoring. Device **1**(4), 100112 (2023). <https://doi.org/10.1016/j.device.2023.100112>
13. L. Zheng, R. Xu, J. Zhang, F. Yu, C. Li et al., Enhanced electrochemical performance by structural design of electrolyte surface combining 3D printing technology with multi-physical modelling. Chem. Eng. J. **451**, 139038 (2023). <https://doi.org/10.1016/j.cej.2022.139038>
14. L. Yuan, T. Zhao, J. Dai, L. Xue, X. Zhang et al., High-density, crosstalk-free, flexible electrolyte-gated synaptic transistors array *via* all-photolithography for multimodal neuromorphic computing. Adv. Funct. Mater. **35**(13), 2418052 (2025). <https://doi.org/10.1002/adfm.202418052>
15. M. Xiong, C.-Y. Yang, J. Ji, A.S. Caravaca, Q. Guo et al., A photo-patternable solid-state electrolyte for high-performance, miniaturized, and implantable organic electrochemical transistor-based circuits. Adv. Mater. **37**(44), e09314 (2025). <https://doi.org/10.1002/adma.202509314>
16. L. Tang, X. Zheng, M. Sun, X. Ren, W. Huang et al., Photopatternable gel electrolytes for stretchable solid-state organic electrochemical transistors. Sci. China Mater. **68**(9), 3212–3218 (2025). <https://doi.org/10.1007/s40843-025-3429-x>
17. T. Li, Z. Qu, J. Si, Y. Lee, V.K. Bandari et al., Monolithically integrated solid-state vertical organic electrochemical transistors switching between neuromorphic and logic functions. Sci. Adv. **11**(11), eadt5186 (2025). <https://doi.org/10.1126/sciadv.adt5186>
18. Q.-G. Chen, W.-T. Liao, R.-Y. Li, I. Sanjuán, N.-C. Hsiao et al., Organic solid-state electrolyte synaptic transistors with photoinduced thiol–ene cross-linked polymer electrolytes for deep neural networks. ACS Materials Lett. **7**(2), 682–691 (2025). <https://doi.org/10.1021/acsmaterialslett.4c02511>
19. Y.J. Jo, Y.-S. Ye, S. Choi, J.H. Shin, J.U. Kim et al., Choline ionic liquid for long-term stable organic electrochemical transistors. Adv. Mater. **38**(4), e12163 (2026). <https://doi.org/10.1002/adma.202512163>
20. M. Li, W. Zhang, X. Lv, X. Liang, M. Wang et al., Regionally controlled ion-doping of organic electrochemical transistors for computing-memory co-integrated neuromorphic systems. npj Flex. Electron. **10**, 11 (2026). <https://doi.org/10.1038/s41528-025-00511-7>
21. Q. Xu, J. Chen, Y. Li, J. Qiu, X. Liu et al., Fully printed dual-gate organic electrochemical synaptic transistor with neurotransmitter-mediated plasticity. IEEE Electron Device Lett. **45**(1), 104–107 (2024). <https://doi.org/10.1109/LED.2023.3335970>
22. J. Ko, X. Wu, A. Surendran, B.T. Muhammad, W.L. Leong, Self-healable organic electrochemical transistor with high transconductance, fast response, and long-term stability. ACS Appl. Mater. Interfaces **12**(30), 33979–33988 (2020). <https://doi.org/10.1021/acsami.0c07913>
23. B. Sun, S.F. Wan Muhamad Hatta, N. Soin, M.F.Z. Bin Abdul Kadir, F.A. Md Rezali et al., Development of screen-printed biodegradable flexible organic electrochemical transistors enabled by poly(3, 4-ethylenedioxythiophene) polystyrene sulfonate and a solid-state chitosan polymer electrolyte. ACS Appl. Electron. Mater. **6**(4), 2336–2348 (2024). <https://doi.org/10.1021/acsaelm.3c01823>
24. M. Li, W. Feng, Y. Lan, Y. Sun, P. Li et al., Effects of selenium incorporation on the performance of polythiophene based organic electrochemical transistors. J. Mater. Chem. C **12**(22), 7935–7942 (2024). <https://doi.org/10.1039/D4TC01226G>
25. A. Makhinia, L. Bynens, A. Goossens, J. Deckers, L. Lutsen et al., Toward sustainability in all-printed accumulation mode organic electrochemical transistors. Adv. Funct. Mater. **34**(28), 2314857 (2024). <https://doi.org/10.1002/adfm.202314857>
26. Y. Zhong, P.D. Nayak, S. Wustoni, J. Surgailis, J.Z. Parrado Agudelo et al., Ionic liquid gated organic electrochemical transistors with broadened bandwidth. ACS Appl. Mater. Interfaces **16**(45), 61457–61466 (2024). <https://doi.org/10.1021/acsami.3c11214>
27. J. Guo, L.Q. Flagg, D.K. Tran, S.E. Chen, R. Li et al., Hydration of a side-chain-free n-type semiconducting ladder polymer driven by electrochemical doping. J. Am. Chem. Soc. **145**(3), 1866–1876 (2023). <https://doi.org/10.1021/jacs.2c11468>
28. X. Wu, Q. He, Z. Zhou, T.L.D. Tam, C. Tang et al., Stable n-type perylene derivative ladder polymer with antiambipolarity for electrically reconfigurable organic logic gates. Adv. Mater. **36**(31), e2308823 (2024). <https://doi.org/10.1002/adma.202308823>
29. W. Yang, K. Feng, S. Ma, B. Liu, Y. Wang et al., High-performance n-type polymeric mixed ionic-electronic conductors: the impacts of halogen functionalization. Adv. Mater. **36**(4), 2305416 (2024). <https://doi.org/10.1002/adma.202305416>
30. I.C. Kwak, Y. Lee, M.J. Kim, Y.J. Choi, D.G. Roe et al., Solid-state homojunction electrochemical transistors and logic gates on plastic. Adv. Funct. Mater. **33**(13), 2211740 (2023). <https://doi.org/10.1002/adfm.202211740>
31. S. Cong, J. Chen, L. Wang, L. Lan, Y. Wang et al., Donor functionalization tuning the N-type performance of donor–acceptor copolymers for aqueous-based electrochemical devices. Adv. Funct. Mater. **32**(29), 2201821 (2022). <https://doi.org/10.1002/adfm.202201821>
32. A.F. Paterson, H. Faber, A. Savva, G. Nikiforidis, M. Gedda et al., On the role of contact resistance and electrode modification in organic electrochemical transistors. Adv. Mater. **31**(37), 1902291 (2019). <https://doi.org/10.1002/adma.201902291>
33. J. Surgailis, A. Savva, V. Druet, B.D. Paulsen, R. Wu et al., Mixed conduction in an N-type organic semiconductor in the absence of hydrophilic side-chains. Adv. Funct. Mater. **31**(21), 2010165 (2021). <https://doi.org/10.1002/adfm.202010165>
34. H.-Y. Wu, C.-Y. Yang, Q. Li, N.B. Kolhe, X. Strakosas et al., Influence of molecular weight on the organic electrochemical transistor performance of ladder-type conjugated polymers. Adv. Mater. **34**(4), 2106235 (2022). <https://doi.org/10.1002/adma.202106235>
35. Y. Li, E.J. Fuller, S. Asapu, S. Agarwal, T. Kurita et al., Low-voltage, CMOS-free synaptic memory based on LiXTiO_2_ redox transistors. ACS Appl. Mater. Interfaces **11**(42), 38982–38992 (2019). <https://doi.org/10.1021/acsami.9b14338>
36. R.D. Nikam, M. Kwak, J. Lee, K.G. Rajput, W. Banerjee et al., Near ideal synaptic functionalities in Li ion synaptic transistor using Li3POxSex electrolyte with high ionic conductivity. Sci. Rep. **9**, 18883 (2019). <https://doi.org/10.1038/s41598-019-55310-8>
37. D. Xie, X. Liang, D. Geng, Q. Wu, C. Liu, An enhanced synaptic plasticity of electrolyte-gated transistors through the tungsten doping of an oxide semiconductor. Electronics **13**(8), 1485 (2024). <https://doi.org/10.3390/electronics13081485>
38. J.H. Park, Y.S. Rim, Liquid–solid interface engineering of ultrathin and solution-processed indium oxide-based electrolyte-gated transistors by gallium doping. ACS Appl. Electron. Mater. **6**(2), 1181–1188 (2024). <https://doi.org/10.1021/acsaelm.3c01563>
39. H. Kwak, C. Lee, C. Lee, K. Noh, S. Kim, Experimental measurement of ungated channel region conductance in a multi-terminal, metal oxide-based ECRAM. Semicond. Sci. Technol. **36**(11), 114002 (2021). <https://doi.org/10.1088/1361-6641/ac25c8>
40. Y. Jeong, H. Lee, D.G. Ryu, S.H. Cho, G. Lee et al., Elucidating ionic programming dynamics of metal-oxide electrochemical memory for neuromorphic computing. Adv. Electron. Mater. **7**(8), 2170034 (2021). <https://doi.org/10.1002/aelm.202170034>
41. J. Cui, F. An, J. Qian, Y. Wu, L.L. Sloan et al., CMOS-compatible electrochemical synaptic transistor arrays for deep learning accelerators. Nat. Electron. **6**(4), 292–300 (2023). <https://doi.org/10.1038/s41928-023-00939-7>
42. L. Zhang, S. Chen, S. Fu, S. Han, L. Zhang et al., Wet etching-based WO_3_ patterning for high-performance neuromorphic electrochemical transistors. Electronics **14**(6) , 1183 (2025). <https://doi.org/10.3390/electronics14061183>
